# The Cancer Patient Empowerment Program: A Comprehensive Approach to Reducing Psychological Distress in Cancer Survivors, with Insights from a Mixed-Model Analysis, Including Implications for Breast Cancer Patients

**DOI:** 10.3390/cancers16193373

**Published:** 2024-10-02

**Authors:** Gabriela Ilie, Gregory Knapp, Ashley Davidson, Stephanie Snow, Hannah M. Dahn, Cody MacDonald, Markos Tsirigotis, Robert David Harold Rutledge

**Affiliations:** 1 Department of Community Health and Epidemiology, Dalhousie University, Halifax, NS B3H 4R2, Canada; 2 Department of Urology, Dalhousie University, Halifax, NS B3H 4R2, Canada; 3 Department of Radiation Oncology, Dalhousie University, Halifax, NS B3H 4R2, Canadarob.rutledge@nshealth.ca (R.D.H.R.); 4 Division of General Surgery, Dalhousie University, Halifax, NS B3H 4R2, Canada; 5 Division of Medical Oncology, Department of Medicine, Dalhousie University, Halifax, NS B3H 4R2, Canada

**Keywords:** psychological distress, cancer survivorship, multifaceted intervention, CancerPEP, randomized clinical trial, mental health, breast cancer, patient empowerment

## Abstract

**Simple Summary:**

Cancer patients frequently encounter significant emotional and psychological challenges that can adversely affect their overall well-being and treatment outcomes. To address these challenges, we developed the Cancer Patient Empowerment Program (CancerPEP), a comprehensive, home-based intervention that incorporates physical exercise, nutritional guidance, and social support. CancerPEP builds on the success of the Prostate Cancer Patient Empowerment Program (PC-PEP), which has demonstrated positive effects on psychological well-being in prostate cancer patients. In this study, we also evaluated whether adding a Heart Rate Variability (HRV) biofeedback device could enhance the effectiveness of CancerPEP. Our findings indicate that while CancerPEP significantly reduced psychological distress and improved emotional well-being across cancer patients—including a breast cancer subgroup—at the end of the intervention (the 6-month point), the addition or lack of an HRV device did not significantly contribute to these improvements for the full sample. These results suggest that CancerPEP, similar to PC-PEP, is a valuable addition to standard cancer care, providing a holistic approach to support patients both mentally and emotionally. However, the inclusion of the HRV device may not be necessary to achieve these benefits.

**Abstract:**

Background/Objectives: Psychological distress is a significant concern among cancer patients, negatively affecting their quality of life and adherence to treatment. The Cancer Patient Empowerment Program (CancerPEP) was developed as a comprehensive, home-based intervention aimed at reducing psychological distress by incorporating physical activity, dietary guidance, and social support. This study aimed to evaluate the feasibility, accrual and attrition rates, safety, and effectiveness of the CancerPEP intervention, with and without the biofeedback device, on psychological distress from baseline to 6 months, specifically focusing on the effects of group randomization and the difference between pre- and post-intervention results. Methods: This single-site, crossover randomized clinical trial included 104 cancer patients who were randomized to receive the CancerPEP intervention, with or without a Heart Rate Variability (HRV) biofeedback monitor. At 6 months, participants who did not receive the device were allowed to use one until the end of the year, while those who did receive the device were followed up to 12 months. Randomization was stratified by the presence or absence of clinically significant psychological distress and metastatic status. Psychological distress was assessed using the Kessler Psychological Distress Scale (K10) at baseline, 6 months, and 12 months. The primary endpoint was the presence of nonspecific psychological distress, as measured by the K10 scale at 6 months from the trial start, based on group randomization. A secondary exploratory analysis assessed psychological distress at baseline, 6 months, and 12 months for both groups, while controlling for group randomization and prognostic covariates. Prognostic covariates included age; comorbidities; time between diagnosis and randomization; treatment modality; relationship status; and use of prescribed medications for anxiety, depression, or both. An exploratory sub-analysis was conducted for the breast cancer subgroup, based on the sample size available after recruitment. The trial is registered at ClinicalTrials.gov (NCT05508412). Results: The provision of the HRV biofeedback monitor in conjunction with the CancerPEP intervention did not significantly affect the primary outcome in either the full sample or the breast cancer subgroup, indicating that the HRV biofeedback provision was not beneficial in this trial. No self-reported or otherwise discovered adverse events at the 6-month mark were observed. About 10% of participants were lost to follow-up in both the early and late HRV monitor provision groups. Participation in the CancerPEP program led to a significant reduction in psychological distress over time. The odds of psychological distress were significantly higher at the start of the trial than at the end of the intervention (aOR = 2.64, 95% CI: 1.53–4.56) or 6 months after the intervention (aOR = 2.94, 95% CI: 1.62–5.30). Similarly, in the breast cancer subgroup, distress was higher at the trial’s start than at 6 months, i.e., after the intervention (aOR = 2.25, 95% CI: 1.24–4.08), or at the end of the trial at 12 months (aOR = 2.73, 95% CI: 1.35–5.52). Conclusions: CancerPEP significantly reduces psychological distress in cancer patients, with consistent improvements noted across various cancer types and stages, including benefits specifically for breast cancer patients. These findings build upon the success of the Prostate Cancer Patient Empowerment Program (PC-PEP), indicating that a similar comprehensive intervention can be advantageous for all cancer patients and may be further tailored to address specific needs. With its holistic approach—encompassing physical, dietary, and psychosocial support—CancerPEP shows promise as a vital component of survivorship care. Ongoing 24-month evaluations will yield critical data on its long-term benefits. Additionally, a randomized trial with a control group (usual care without intervention) for breast cancer patients is currently under way and could potentially guide the integration of CancerPEP into standard oncology care to enhance patient outcomes and quality of life.

## 1. Introduction

Cancer patients worldwide face not only the physical burdens of their disease but also significant mental health challenges, with psychological distress emerging as a critical concern. Depression, anxiety, and distress are prevalent among cancer patients, severely diminishing their quality of life and potentially impairing treatment adherence. A systematic review by Ikhile et al. [1] reported that approximately 25–30% of cancer patients experience clinically significant symptoms of depression and anxiety throughout their cancer journey, from diagnosis to treatment and survivorship [1]. In Canada, cancer remains the leading cause of death, and the number of new cases and cancer-related deaths is projected to rise as the population ages [2].

Nearly half of all cancer patients in Canada are estimated to experience a mental health disorder at some point during their illness, underscoring the profound psychological impact of cancer [3]. These mental health challenges can persist long after diagnosis and treatment, leading to poorer treatment adherence, increased health care utilization, and worse survival outcomes [4,5].

The onset of depression and anxiety in cancer patients is influenced by a range of biopsychosocial and sociodemographic factors that vary throughout the cancer continuum. Ikhile et al. [1] identified 32 distinct risk factors for mental health deterioration in cancer patients, clustered into cancer-specific, biological, psychological, and social domains. Notably, over half of these risk factors (60%) were psychosocial, highlighting the critical importance of addressing both the psychological and social dimensions in cancer care. Psychological risk factors, such as low social support, emotional suppression, and maladaptive coping mechanisms, constituted 41% of the total, while 19% were related to social factors, including living alone, low socioeconomic status, and limited engagement in community activities [1]. Understanding these risk factors is essential for developing personalized interventions to mitigate mental health disorders in cancer patients.

Despite the increasing recognition of the need to address psychological distress in cancer patients, there is a scarcity of randomized clinical trials evaluating comprehensive interventions that target distress across multiple cancer types. Many existing survivorship programs are limited in scope and fail to address the full spectrum of physical and psychosocial challenges faced by cancer survivors. Murnaghan et al. [6] emphasized that many interventions inadequately target outcomes critical to survivors, such as reintegration into daily life after treatment [6]. This gap underscores the need for holistic, personalized approaches in survivorship care.

The Cancer Patient Empowerment Program (CancerPEP) was developed in response to this need. This structured, home-based intervention is designed to meet the educational, physical, and psychosocial needs of cancer patients throughout their treatment journey, with the primary aim of reducing psychological distress. The success of its predecessor, the Prostate Cancer Patient Empowerment Program (PC-PEP), demonstrated the potential of such interventions (PCPEP.org) [7]. PC-PEP, a 6-month home-based program, significantly improved mental health; self-efficacy; and management of treatment-related side effects, such as urinary and sexual dysfunction, in prostate cancer patients [7,8,9,10,11,12]. A randomized controlled trial (RCT) of PC-PEP showed substantial reductions in psychological distress and improved oncological outcomes [7,8,9,10].

However, the PC-PEP program highlighted areas for improvement. Specifically, the Heart Rate Variability (HRV) biofeedback monitor, part of the stress-reduction component, received lower participant ratings [7]. Subsequent analyses revealed that, in contrast to the significant improvements in self-efficacy and illness perceptions, heart rhythm coherence did not change meaningfully after the intervention [8]. This may be attributed to the program’s complexity and lower adherence to the HRV training component, suggesting that participants benefited more from other elements such as physical activity, psychosocial support, and dietary changes. These findings raise questions about the necessity of including the HRV device in future iterations, as its exclusion may simplify the program and improve cost-effectiveness [8]. Despite this, a cost-effectiveness analysis of PC-PEP demonstrated significant savings, with an estimated reduction of 660.89 CAD per patient at 12 months, while preventing a significant proportion of potential cases of psychological distress [7]. These findings highlight the need to evaluate the contribution of each program component to optimize its integration into standard cancer care.

Building on the success and lessons learned from PC-PEP, we developed CancerPEP, an enhanced intervention tailored for a broader range of cancer patients, and assessed its feasibility, accrual and attrition rates, and participants’ safety. CancerPEP incorporates dietary education, healthy living habits, and attitudinal healing techniques delivered over a 26-week period. Given the substantial cost associated with the HRV biofeedback monitor (approximately 200 CAD per device), we employed a randomized trial approach to assess whether its inclusion meaningfully contributes to the primary outcome of psychological distress and the need for clinical treatment at the 6-month follow-up [7].

The primary endpoint was the presence of nonspecific psychological distress, measured using the Kessler Psychological Distress Scale (K10) at 6 months from the trial start, based on group randomization. The study assessed the intervention’s impact at discrete time points (baseline, 6 months, and 12 months), controlling for group randomization and prognostic covariates, including age; comorbidities; time between diagnosis and randomization; treatment modality; relationship status; and prescribed medications for anxiety, depression, or both.

Additionally, if group randomization was found not to affect the primary endpoint at 6 or 12 months when assessed against the baseline, we aimed to explore the longitudinal effects of CancerPEP on psychological distress, irrespective of group randomization, to examine changes immediately and 6 months after intervention (at the 12-month mark). Based on the sample size available after recruitment, an exploratory sub-analysis was conducted for the breast cancer subgroup. This study seeks to clarify the role of the HRV monitor and to provide critical insights into improving mental health outcomes in cancer patients through comprehensive interventions.

## 2. Materials and Methods

This single-site, university-based, tertiary-care, crossover randomized clinical trial assessed the eligibility of 127 self-reported cancer patients. Participants were either referred by their oncologist or self-referred through poster advertisements distributed to various non-profit cancer support organizations across Canada, including Nova Scotia Cancer Care. Recruitment took place over a two-week period in December 2022. The full study protocol is provided in the Supplementary Materials.

Eligible participants were required to be 18 years or older, have a documented history of any type or stage of cancer, and have an estimated life expectancy of more than one year. Medical clearance to engage in low- to moderate-intensity exercise, including light strength training, was also necessary. Additionally, participants needed to demonstrate proficiency in reading English and have consistent access to email and the internet, as these were required for participation in the six-month intervention. Participants were also required to complete online quality-of-life assessments at baseline, 6 months, 12 months, and 24 months. Informed consent was obtained using a study-specific consent form approved by the Nova Scotia Health Authority (protocol number 1028421; ClinicalTrials.gov NCT05508412).

**Figure 1:**
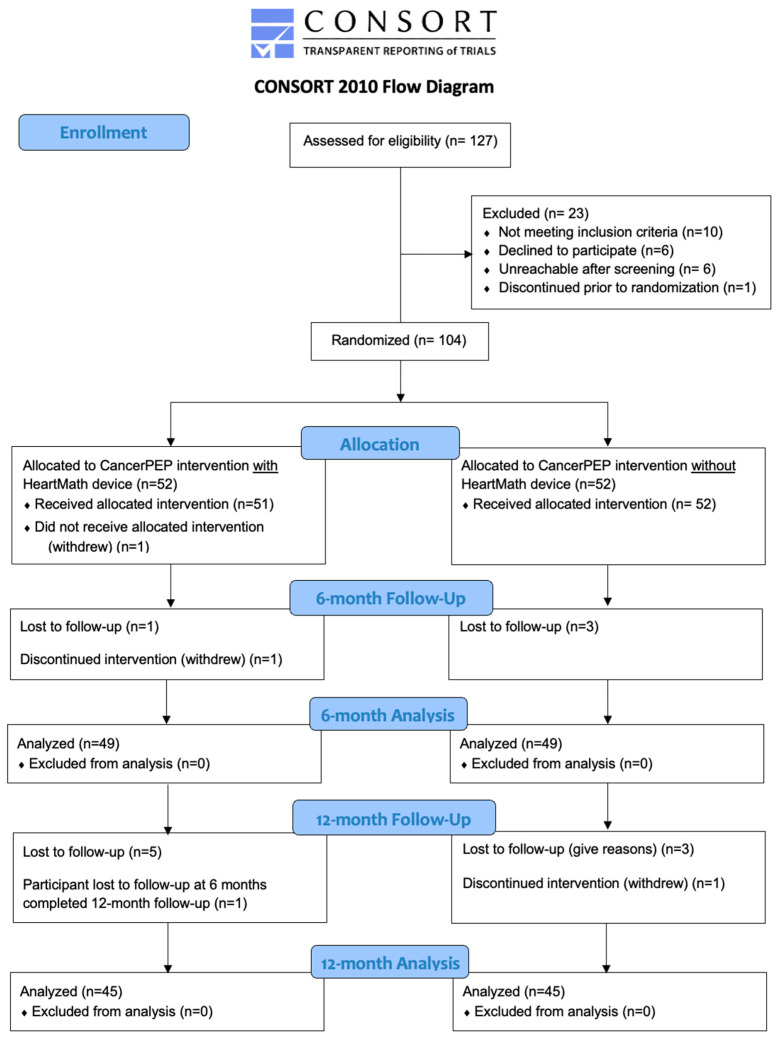
CONSORT 2010 flow diagram. CONSORT = Consolidated Standards of Reporting Trials; CancerPEP = Cancer Patient Empowerment Program.

The trial adhered to the Consolidated Standards of Reporting Trials (CONSORT) guidelines (see Supplemental Material). Of the 127 patients assessed for eligibility, 104 met the inclusion criteria and were randomized. One participant withdrew consent immediately after enrollment, another withdrew during the six-month intervention, and one more before the 12-month intervention (Figure 1). Participants were randomized in a 1:1 ratio to either the CancerPEP intervention with a HeartMath [11] Heart Rate Variability (HRV) monitor or the intervention without the HRV monitor, with participants in the latter group given the option to access the HRV monitor after the initial six-month period.

Randomization was conducted using a computer-generated, fixed block allocation scheme, following the method of Zelen [12], to ensure balance with respect to two key factors: the presence or absence of nonspecific psychological distress (Kessler Psychological Distress Scale [K10]; scores ≥20 or <20) and the presence of metastatic disease (yes/no). The randomization table was securely stored in a password-protected file , accessible only to the principal investigator (PI), who was not involved in the consent or assessment processes. Patients, clinicians, and research staff were blinded to the randomization process. Following the completion of the baseline online quality-of-life survey, participants were assigned to their respective intervention groups based on the pre-determined randomization sequence (see Supplemental Material).

Of the 104 eligible participants, 52 were randomized to the CancerPEP intervention with the HeartMath HRV monitor, while the remaining 52 were assigned to the intervention without the monitor. After the initial six-month period, participants in the latter group were offered the HeartMath HRV monitor for an additional six months. All participants had ongoing access to CancerPEP materials, as well as monthly live online videoconferences, beyond the initial six-month intervention period.

Participants completed health-related quality-of-life surveys online at baseline, 6 months, and 12 months, assessing both primary and secondary outcomes, as well as relevant prognostic covariates. The trial is ongoing, with 24-month follow-up data expected to be available after December 2024.

### 2.1. Exposure

Details of the CancerPEP intervention are fully described in the study protocol (see Supplementary Materials) and at CancerPEP.org. In brief, participants received daily emails for six months, each containing a 3- to 5-min video presented by coauthors G.I. and R.D.H.R. These videos provided education, motivation, and guidance for daily physical, mental, and social activities. Each activity was supported by additional video links demonstrating the components, which were organized into a structured weekly schedule. Participants were encouraged to engage in daily exercise, including resistance training twice weekly using provided elastic exercise bands. Exercise routines were tailored to participants’ fitness levels, with four intensity levels ranging from beginner to expert. Strength yoga routines were similarly customized, with three intensity levels: mild, intermediate, and advanced.

Participants were instructed to practice daily relaxation techniques using a biofeedback device (HeartMath) designed for stress reduction. Nutritional guidance focused on a diet rich in fruits and vegetables, supported by weekly cooking video demonstrations of plant-based recipes specifically developed for cancer patients. These 26 cooking videos were released sequentially every Saturday and were accessible via an online program link. Time-restricted eating was encouraged, accompanied by guidance on consuming whole foods, avoiding chemicals, and practicing moderation.

The program also addressed healthy habit formation, covering topics such as sleep hygiene, awareness of environmental toxins, and vitamin D intake. Additionally, 26 weekly videos introducing attitudinal healing principles were released every Sunday. These principles explored topics including intimacy, connection, sexuality, communication techniques, and relationship enhancement.

To foster social support, participants were encouraged to maintain frequent, meaningful connections with loved ones. Optional support activities included weekly calls with two fellow participants and participation in monthly live Zoom videoconferences. These videoconferences featured a 20-min scientific presentation by the program leads, covering key aspects of the intervention, practical tools for patient activation, and the latest scientific evidence. Following the presentation, participants engaged in breakout discussions and large-group sharing sessions. Partners were invited to attend and often joined partner-only breakout sessions.

### 2.2. Outcomes

The primary outcome was the level of nonspecific psychological distress, assessed using the Kessler Psychological Distress Scale (K10) at baseline, 6 months, and 12 months. The K10 is a widely used 10-item instrument designed to evaluate psychological distress experienced over the past 30 days, with total scores ranging from 10 to 50 [13]. It includes two subscales: depression (items 1, 4, 7, 8, 9, and 10) and anxiety (items 2, 3, 5, and 6). A score of 20 or higher indicates significant distress (coded as 1), while a score below 20 indicates its absence [14]. In this study, the K10 demonstrated excellent internal consistency, with Cronbach’s α values of 0.89, 0.86, and 0.87 at baseline, 6 months, and 12 months, respectively. These values align with previous research, as the K10 is known for its robust psychometric properties and its utility in screening for internalizing disorders [15,16].

### 2.3. Prognostic Covariates

Several prognostic covariates were included in the primary analysis due to their established influence on mental health outcomes in cancer patients. These covariates comprised patient age (in years) [17], the number of comorbidities [18], the time elapsed between diagnosis and study randomization [19,20], and the treatment modality received to date for their cancer diagnosis. Treatment modalities were categorized as follows: (1) surgery with or without radiation, hormone therapy (endocrine therapy), and/or chemotherapy; (2) primary radiotherapy with or without hormone therapy (endocrine therapy) and/or chemotherapy; and (3) chemotherapy with or without hormone therapy [21]. Additionally, relationship status was coded as 1 for “yes” and 0 for “no” [22], while the use of prescribed medications for depression, anxiety, or both was coded as 1 for present and 0 for absent [23]. These variables were identified a priori for inclusion as covariates in the primary analysis.

### 2.4. Sample Size Calculation

The sample size calculation was based on detecting a statistically significant difference in the incidence of nonspecific psychological distress (K10 score ≥ 20) between the Early HRV (CancerPEP with the use of an HRV biofeedback device) and Late HRV (CancerPEP without the HRV device initially, but with the option to use it after the intervention for 6 months) groups at the 6-month follow-up. Assuming a two-sided test with a significance level (α) of 0.05, a power of 0.80, and an expected incidence of psychological distress of 30% in the group not receiving the HRV monitor versus 10% in the group receiving the monitor, a total sample size of 100 participants was deemed necessary to detect a meaningful effect. This calculation was informed by previous research indicating that approximately 30% of cancer patients experience significant psychological distress during or several years after their treatment [18]. The sample size ensures sufficient power to detect clinically relevant differences in distress outcomes between the two groups, accounting for potential loss to follow-up and ensuring the robustness of the study’s conclusions [24,25].

### 2.5. Statistical Analysis

To address the aims of the study, we first evaluated the feasibility of the CancerPEP intervention by assessing accrual and attrition rates among participants. Feasibility was measured by the number of participants successfully recruited for the trial, while accrual was calculated as the percentage of eligible individuals who consented to participate. We monitored attrition by tracking the number of participants who completed the study versus those who dropped out, and we defined drop-out rates as the percentage of participants lost to follow-up at each time point.

Participant safety was assessed by recording any self-reported adverse events or otherwise discovered adverse effects throughout the study duration. This information was collected during scheduled follow-up assessments and was crucial for evaluating the safety profile of the CancerPEP intervention.

Baseline characteristics, including demographic, clinical, and treatment-related variables, were compared between the two groups: Early HRV (CancerPEP with the use of an HRV biofeedback device) and Late HRV (CancerPEP without the HRV device initially, but with the option to use it after the intervention for 6 months). Continuous variables are reported as medians and interquartile ranges (IQRs), while categorical variables are presented as counts and percentages. The Mann–Whitney U test was used for comparing continuous variables, while the chi-square test or Fisher’s exact test was applied for categorical variables as appropriate. All tests were two-tailed, with a significance level set at *p* < 0.05.

To evaluate the primary endpoint—the presence of nonspecific psychological distress at 6 months—we applied two multiple logistic regression models to assess the association between the timing of HRV device provision (early vs. late) and the likelihood of psychological distress. These models were adjusted for baseline K10 scores and relevant prognostic covariates [26,27]. The analyses were performed separately for the full cohort and the breast cancer subgroup, reporting adjusted odds ratios (aORs) and 95% confidence intervals (CIs).

In our study, we initially set out to determine whether group randomization (i.e., whether participants received the HRV biofeedback monitor early or late) influenced the primary endpoint of psychological distress at 6 or 12 months when compared to baseline measurements. If the analysis indicated that group randomization did not significantly affect the primary outcome, we would then shift our focus to investigating the longitudinal effects of the CancerPEP intervention on psychological distress, regardless of group assignment. This approach allowed us to assess changes in psychological distress at two key time points: immediately after the intervention (at 6 months) and again at 12 months after the start of the intervention.

To quantify the impact of the CancerPEP intervention over time on psychological distress, we utilized Generalized Estimating Equations (GEE) models, which allowed us to analyze data collected at three distinct time points: baseline, 6 months, and 12 months. These models employed a binomial distribution with a logit link function to assess the likelihood of psychological distress (defined as a K10 score of 20 or higher) while accounting for correlations between repeated measures from the same participants. We specified an exchangeable working correlation matrix to address these within-subject correlations.

For accurate parameter estimation, we used a robust covariance matrix, which helped ensure that our standard errors were reliable. The effects of the model were assessed using Wald chi-square tests, making adjustments for various prognostic covariates, such as age, treatment modality, relationship status, comorbidities, the duration from diagnosis to study enrollment, and the use of medications for anxiety or depression [26,27]. Additionally, we conducted separate analyses for the overall cohort and the breast cancer subgroup to gain deeper insights into the intervention’s effects across different populations.

An interaction term between time and group was initially included in the GEE models to explore whether the intervention’s effect varied across time points. However, the interaction was not statistically significant, leading to the decision to conduct the main GEE analyses without the interaction term to enhance clarity. Results from the interaction term analysis are provided in the supplementary materials.

In addition to the Generalized Estimating Equations (GEE) analyses, we employed mixed-model analyses to further investigate the effects of the CancerPEP intervention on psychological distress. The use of mixed models was particularly important in this context because they allow the examination of individual variability among participants, which is crucial when assessing interventions that may have different impacts on different individuals.

The mixed model tested for the main effects of group randomization and time, incorporating an interaction term to evaluate whether the intervention’s effects varied across time points. Although this interaction was not statistically significant, the decision to rerun the model without it helped simplify the analysis and reduce unnecessary complexity. This focus on the main effect of time ensures clarity in interpreting the findings while still accounting for variations in the data.

One of the key advantages of mixed models is their ability to accommodate a hierarchical data structure, where repeated measures are taken from the same individuals over time. This approach enables us to analyze both fixed effects (overall trends associated with time) and random effects (individual variations), providing a more nuanced understanding of how the CancerPEP intervention influences psychological distress across the participant population.

All statistical tests were two-tailed, with significance set at *p* < 0.05. Pairwise comparisons were performed to evaluate estimated marginal mean differences across time points. All analyses were conducted using IBM SPSS statistical software, version 29.0 [28].

## 3. Results

### 3.1. Feasibility and Accrual

The trial successfully recruited a total of 104 cancer patients accrued within two weeks of trial opening, with participants being randomly assigned to two groups: Early HRV (CancerPEP with the use of an HRV biofeedback device) and Late HRV (CancerPEP without the HRV device initially, but with the option to use it after the intervention for 6 months). The recruitment process demonstrated strong feasibility, as the study met its target enrollment within the specified timeline.

### 3.2. Attrition

At the 12-month follow-up, approximately 10% of participants were lost to follow-up in both the early and late HRV monitor provision groups. This attrition rate highlights the challenges in maintaining participant engagement throughout the trial, although the overall retention was relatively strong considering the duration of the study (Figure 1).

### 3.3. Safety

Safety assessments revealed that there were no adverse events reported during the trial. Participants reported no serious adverse events related to the CancerPEP intervention or the HRV biofeedback device, affirming the safety of the intervention throughout the study period.

### 3.4. Baseline Characteristics

The baseline characteristics of the two groups were generally comparable (Table 1). However, significant differences were observed in age (*p* = 0.013), with the Early HRV group consisting of older participants than the Late HRV group. Additionally, there was a significant difference in living situation (*p* = 0.047), as participants in the Early HRV group were more likely to reside in urban areas. All other baseline characteristics were similar between the groups.

**Figure 2:**
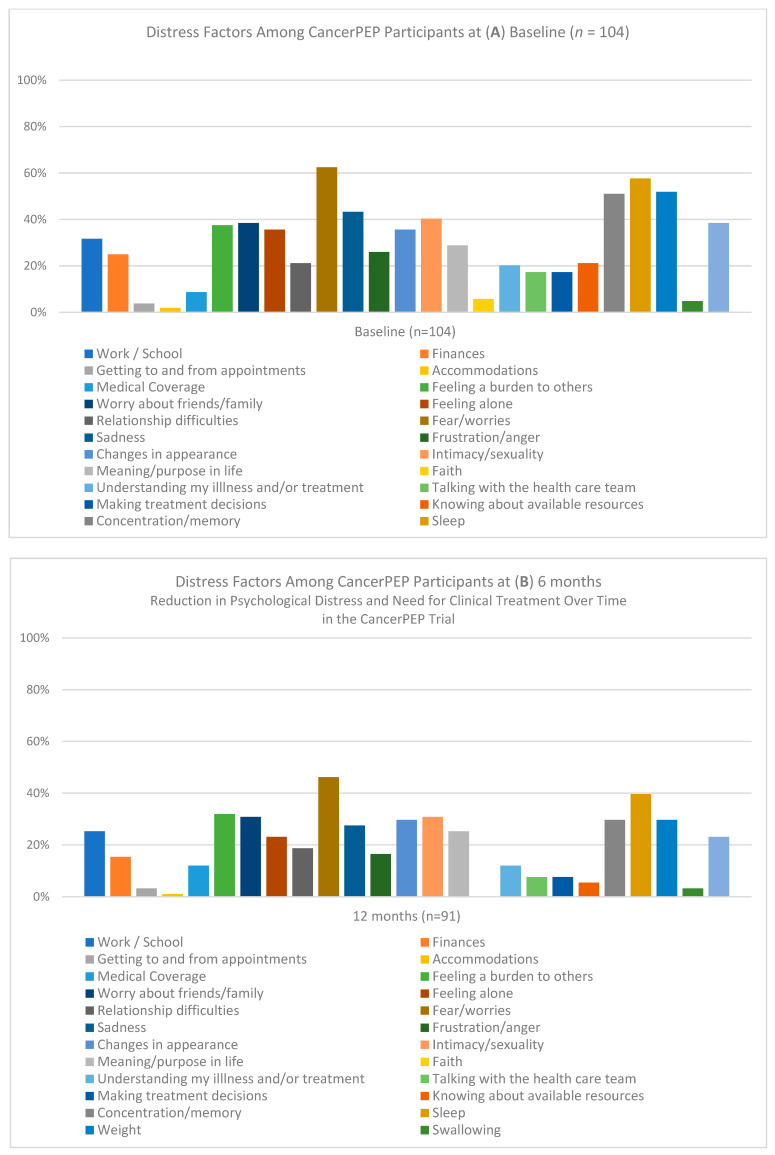

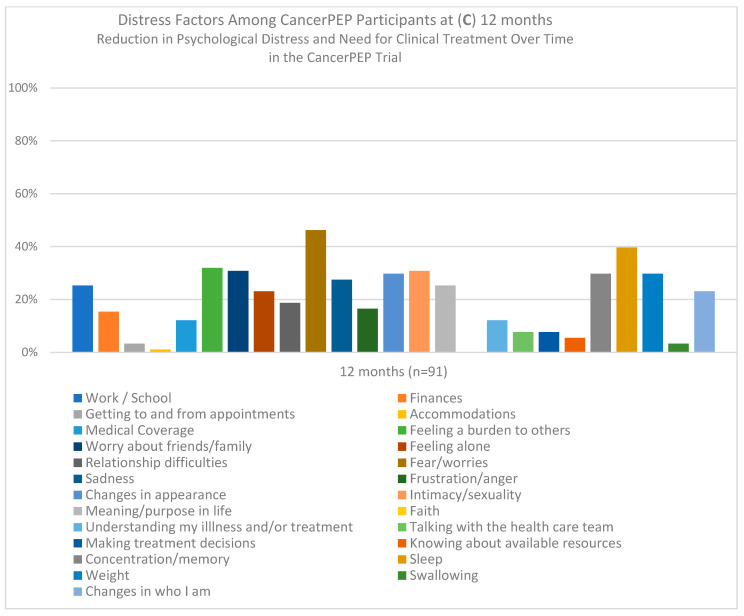
Distress factors among CancerPEP participants at baseline (**A**), 6 months (**B**), and 12 months (**C**). This figure illustrates the percentage of CancerPEP participants reporting various distress factors at three key time points during the trial: baseline (*n* = 104), 6 months (*n* = 102), and 12 months (*n* = 91). The distress factors assessed include work/school concerns, finances, feeling alone, relationship difficulties, understanding illness, and others. The figure demonstrates changes in the prevalence of these distress factors over time, reflecting the potential impact of the CancerPEP program on reducing psychological distress among participants.

In addition to baseline characteristics, distress factors reported by participants at baseline, 6 months, and 12 months were evaluated, as presented in Figure 2. This figure illustrates the percentage of participants reporting various distress factors throughout the trial. At baseline, the most reported sources of distress included concerns related to work/school, finances, and feeling like a burden to others. By the 6- and 12-month follow-ups, many of these distress factors had notably decreased, suggesting potential beneficial effects of the CancerPEP program on participants’ psychological well-being. Specifically, the percentage of participants reporting distress related to work/school showed a significant decline from baseline to 12 months. Additionally, other factors, such as concerns related to faith and understanding their illness, displayed variations over time, further indicating the program’s potential impact on reducing psychological distress.

### 3.5. Logistic Regression Analysis

At 6 months, 18% of patients in the early HRV biofeedback group and 29% in the late HRV biofeedback group screened positive (K10 score ≥ 20) for psychological distress and the need for clinical treatment, compared to baseline rates of 39% and 46%, respectively. By 12 months, these rates were 20% in the Early HRV group and 23% in the Late HRV group.

Table 2 presents the results of multiple logistic regression analyses, which assessed the impact of group randomization (HRV device provision) on psychological distress at 6 and 12 months, adjusted for baseline distress and other prognostic covariates. Group randomization did not have a statistically significant effect on participants’ psychological distress in the full cohort (6 months: aOR = 0.72, 95% CI: 0.19–2.69, *p* = 0.6; 12 months: aOR = 1.14, 95% CI: 0.30–4.39, *p* = 0.8) or in the breast cancer cohort (6 months: aOR = 0.39, 95% CI: 0.05–3.13, *p* = 0.4; 12 months: aOR = 0.24, 95% CI: 0.03–1.98, *p* = 0.19). These results indicate that the provision of the HRV device did not significantly impact psychological distress in either the overall sample or the breast cancer subgroup at these time points.

### 3.6. Generalized Estimating Equations (GEE) Analysis for the Full Sample

The Generalized Estimating Equations (GEE) model was employed to assess the association between psychological distress (K10 score ≥ 20) and key covariates, including group randomization and other prognostic factors, across three time points (baseline, 6 months, and 12 months). This approach accounts for the correlated nature of repeated measures within individuals, providing population-averaged estimates. Analyses were conducted separately for both the full cohort and the breast cancer cohort.

Initial models included an interaction term between time and group randomization to evaluate whether the intervention’s effect varied across time points. However, this interaction term did not reach statistical significance in either the full sample (χ²(2) = 0.997, *p* = 0.6) or the breast cancer subsample (χ²(2) = 0.739, *p* = 0.7). Consequently, the interaction term was excluded from the final model to enhance clarity and reduce complexity [29]. 

**Figure 3:**
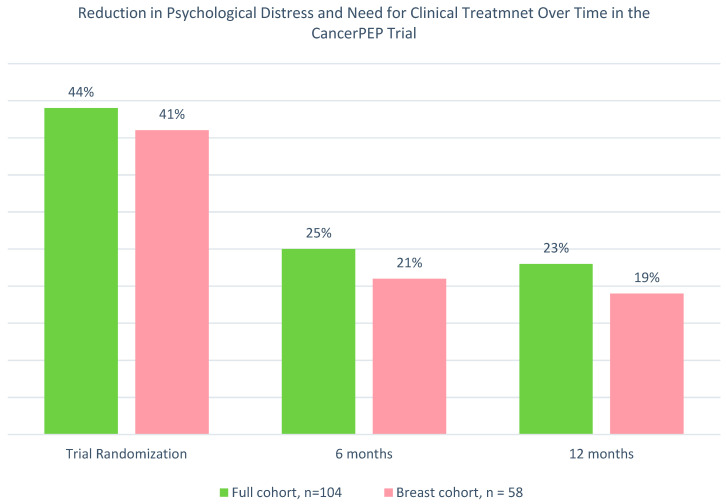
Reduction in psychological distress and need for clinical treatment over time in the CancerPEP trial. The figure illustrates the percentage of participants in the full cohort (*n* = 104) and the breast cancer cohort (*n* = 58) who screened positive for psychological distress (K10 score ≥ 20) and required clinical treatment at three time points: trial randomization (baseline), 6 months, and 12 months. The graph demonstrates a significant reduction in distress over time in both cohorts, with the most substantial decline observed at the 6-month follow-up. The full cohort and breast cancer cohort show similar trends, though the breast cancer cohort exhibits a slightly lower distress percentage at each follow-up point.

The CancerPEP intervention resulted in a significant reduction in psychological distress over time (Figure 3). In the full cohort, distress levels decreased from 44% at baseline to 25% at 6 months and 23% at 12 months. Similarly, in the breast cancer cohort, distress levels decreased from 41% at baseline to 21% at 6 months and 19% at 12 months. These reductions suggest the overall effectiveness of the CancerPEP intervention in reducing psychological distress over time.

Table 3 indicates that time was a significant predictor of psychological distress (Wald χ²(2) = 18.128, *p* < 0.001). Specifically, the odds of psychological distress were significantly higher at baseline than at 12 months (OR = 2.935, 95% CI: 1.624–5.303, *p* < 0.001). However, no significant difference was observed between 6 months and 12 months (OR = 1.112, 95% CI: 0.587–2.107, *p* = 0.744). Additionally, the use of prescribed medication for anxiety or depression was a significant predictor of distress (OR = 3.415, 95% CI: 1.364–8.550, *p* = 0.009), while group randomization and other prognostic covariates were not significant.

### 3.7. GEE Sub-Analysis for the Breast Cancer Cohort

The GEE analysis for the breast cancer cohort, detailed in Table 4, similarly identified time as a significant predictor of psychological distress (Wald χ²(2) = 11.533, *p* = 0.003). The odds of reporting psychological distress were significantly higher at baseline than at 12 months (OR = 2.73, 95% CI: 1.35–5.52, *p* = 0.005). No significant difference was found between 6 months and 12 months (OR = 1.22, 95% CI: 0.56–2.65, *p* = 0.623). Medication use for anxiety or depression was a significant predictor of distress in this cohort (OR = 4.31, 95% CI: 1.16–15.98, *p* = 0.029), while other covariates, such as age and relationship status, were not significant.

### 3.8. Mixed-Model Analysis for the Full Sample

To further investigate the effects of the CancerPEP intervention on psychological distress while accounting for individual variability among participants, we employed mixed-model analyses. This approach allows the examination of both fixed effects (overall trends associated with time) and random effects (individual variations). The overall model was statistically significant (χ²(11) = 38.51, *p* < 0.001). The model included main effects for group randomization and time, along with their interaction term. However, as the interaction was not statistically significant (χ²(2) = 0.88, *p* = 0.6), the decision was made to exclude this term from the final model, enhancing clarity and interpretability.

The revised model (Table 5), without the interaction term, remained significant (χ²(9) = 37.62, *p* < 0.001). The analysis indicated significant changes in psychological distress over time, with participants being significantly less likely to report distress at both 6 months (aOR = 0.37, *p* = 0.004) and 12 months (aOR = 0.34, *p* = 0.002) than at baseline.

Other significant predictors included the use of medication for anxiety or depression, which increased the odds of psychological distress (aOR = 3.4, *p* < 0.001), and a higher number of comorbidities (aOR = 1.4, *p* = 0.041).

### 3.9. Mixed-Model Analysis for the Breast Cancer Subsample

A mixed-model analysis was also performed to assess the longitudinal effects of the CancerPEP intervention on psychological distress within the breast cancer subgroup. The overall model was statistically significant (χ²(11) = 32.01, *p* < 0.001). As with the full sample, the model included main effects for group randomization and time, plus an interaction term, which was again found to be non-significant (χ²(2) = 0.50, *p* = 0.8).

The re-run model (Table 6), without the interaction term, remained significant (χ²(9) = 31.52, *p* < 0.001). This analysis revealed a significant effect of group randomization on psychological distress, with participants receiving the HRV monitor later being 3.11 times more likely to report distress than those in the early HRV monitor group. However, this effect is confounded by the timing of HRV monitor provision, as those in the late group had the device provided after the intervention. This suggests that the apparent impact of group randomization on psychological distress may not solely reflect the differences in intervention effectiveness, but rather the timing of when participants had access to the HRV device. As such, while the main effect of group provides some insights, it is crucial to interpret these findings cautiously, considering that the timing of HRV monitor provision may influence psychological distress independently of the intervention itself. The longitudinal analysis may still provide valuable information regarding trends over time, but the confounding effect of timing could complicate the interpretation of how group assignment influences psychological distress at different follow-up points.

Participants experienced a significant reduction in distress when comparing the 12-month follow-up to baseline (aOR = 0.36), indicating that distress levels decreased substantially over the intervention period. The comparison between 6 months and baseline approached significance (*p* = 0.062), while the difference between 6 months and 12 months was not statistically significant (*p* = 0.8).

Table 7 presents the exit evaluations of the CancerPEP program by participants in both intervention groups at the end of the 6-month program. Overall, participants rated the program highly across various dimensions, with mean scores consistently above 7 on a 0–10 scale, indicating strong positive perceptions of the program’s components.

Participants in both groups rated the perceived competence of the CancerPEP research and clinical team very highly, with mean scores of 9.39 in the group who received the HRV monitor early and 9.13 in the group who received the HRV monitor late, reflecting strong confidence in the team’s ability to deliver the program effectively. Similarly, the likelihood of recommending the CancerPEP program to others was high, with mean scores of 9.41 and 9.17, respectively, indicating overall satisfaction with the program.

Both groups equally emphasized the importance of implementing CancerPEP as part of standard care from the day of diagnosis, with a mean score of 9.00, underscoring the perceived relevance and necessity of the intervention. The perceived overall usefulness of the program was also rated favorably, though slightly lower, with mean scores of 8.57 for the group who received the HRV monitor early and 8.11 for the group who received the HRV monitor late.

A notable difference between the groups was observed in their interest in continuing the program beyond the 6-month period. The group who received the HRV monitor late reported greater interest (77%) than the group who received the HRV monitor early (61%). Furthermore, the group who received the HRV monitor late showed greater interest in becoming mentors for the program, with 34% expressing interest compared to 22% in the group who received the HRV monitor early.

The perceived usefulness of various program components, including strength and aerobic exercises, dietary advice, daily educational videos, and meditation, was generally well rated. However, the group who received the HRV monitor early gave it a lower usefulness score (mean = 4.88), suggesting that participants did not find this particular tool as beneficial as other elements of the program.

In summary, participants from both groups reported high levels of satisfaction with the CancerPEP program, valuing its educational, psychological, and physical health components. The slightly higher interest in program continuation and mentorship in the group who received the HRV monitor late suggests that the inclusion of the HRV device may not have significantly enhanced participants’ engagement or their perceived benefits from the program.

## 4. Discussion

The feasibility of the Cancer Patient Empowerment Program (CancerPEP) was demonstrated through successful adherence to the study protocols and the CONSORT guidelines. Out of 127 patients assessed for eligibility, 104 were randomized, achieving a satisfactory accrual rate for the study. The attrition rate was minimal, with only three participants withdrawing from the study, indicating strong retention throughout the intervention period. Importantly, no adverse events, serious or otherwise, were reported, highlighting the safety of the CancerPEP intervention. These results underscore the point that CancerPEP is not only effective but also practical and safe for implementation in clinical settings.

Building on this foundation of feasibility and safety, this study evaluated the effectiveness of CancerPEP as a comprehensive, home-based intervention aimed at reducing psychological distress among cancer patients. The results demonstrate that CancerPEP significantly reduces psychological distress and the need for clinical treatment over time, as evidenced by the decline in Kessler Psychological Distress Scale (K10) scores from baseline to 6 and 12 months. These findings suggest that CancerPEP is a valuable tool for addressing the mental health needs of cancer patients, particularly in the context of survivorship care.

### 4.1. Effectiveness of CancerPEP in Reducing Psychological Distress

The significant reduction in psychological distress observed in both the full cohort and the breast cancer subsample in the GEE analysis underscores CancerPEP’s overall effectiveness. These results are consistent with prior research, which has shown that structured psychosocial interventions can positively impact mental health outcomes among cancer patients [1,6,30,31]. The substantial reduction in distress by the 6-month mark suggests that the tools and resources provided early in the program are effective across various stages of cancer and survivorship. Regardless of the specific stage of their cancer journey, patients benefit from the structured support and coping strategies that CancerPEP offers, helping them navigate the emotional and psychological challenges associated with both active treatment and long-term survivorship.

Furthermore, the consistent reduction in distress across different cancer types, particularly in the breast cancer sub-sample analysis, highlights CancerPEP’s broad applicability. This finding aligns with prior results from the Prostate Cancer Patient Empowerment Program (PC-PEP), which demonstrated similar effectiveness in reducing psychological distress among prostate cancer patients [7,8]. Together, these results suggest that the PEP programs can be effective across different cancer types, including breast and prostate cancer. The multifaceted approach of these programs—encompassing physical activity, dietary modifications, and psychosocial support—likely contributes to these positive outcomes by addressing multiple facets of well-being [32,33]. Multifaceted interventions, such as CancerPEP, have been recognized as superior to simpler, single-component programs in meeting the diverse needs of cancer patients. For instance, Mundle et al. [33] found that among 22 randomized trials in prostate cancer, only three multifaceted programs improved all three mental health outcomes (distress, depression, and anxiety). Our study extends this literature by demonstrating the effectiveness of a similar comprehensive approach in CancerPEP.

The PEP programs also extend beyond the initial 6-month intervention period through monthly videoconferences, where participants receive scientific updates, engage in discussions, and benefit from mentorship roles. This ongoing engagement enhances the program’s sustainability and facilitates expansion to other medical and cancer non-profit sites [7,33].

Patient activation plays a critical role in CancerPEP’s success by empowering patients to take an active role in their care, complementing the medical system’s efforts [34,35,36,37]. The daily video messages clarify patient responsibilities—such as regular exercise, dietary changes, and social support—thereby fostering a sense of ownership over health outcomes. Research consistently shows that higher levels of patient activation are associated with better health outcomes, improved adherence, and greater engagement in preventive behaviors [34,35,36,37,38]. The credibility of the program’s clinical leads, who are experts in oncology and quality-of-life research, further strengthens patient motivation to adopt healthier behaviors [38]. However, behavior change is complex and influenced by numerous psychological, social, and environmental factors [39,40,41], making CancerPEP’s comprehensive and patient-centered approach particularly valuable.

It is important to note that while our mixed-model analysis revealed a significant effect of group randomization on psychological distress in the breast cancer subsample—indicating that participants receiving the HRV monitor later were 3.11 times more likely to report distress than those in the early HRV group—this effect may be confounded by the timing of HRV monitor provision. This timing discrepancy could inherently influence psychological outcomes, as those receiving the monitor after the intervention may not have benefited from the immediate supportive effects that the program offers. However, this confounding primarily impacts the interpretation of the group randomization effect rather than the validity of the Generalized Estimating Equations (GEE) analysis. The GEE approach focuses on overall trends in psychological distress over time and controls for other covariates, thereby allowing us to capture the longitudinal impact of the CancerPEP intervention across all participants, irrespective of group assignment. Importantly, the results from both GEE and mixed-model analyses revealed consistent trends, reinforcing the robustness of our findings and supporting the program’s effectiveness in reducing psychological distress, independent of the effects of group randomization.

### 4.2. The Role of the HRV Biofeedback Monitor

Despite CancerPEP’s overall success, the Heart Rate Variability (HRV) biofeedback monitor did not significantly enhance the program’s effectiveness in reducing psychological distress at the end of the 6 months CancerPEP intervention. Participants reported lower perceived usefulness of the HRV biofeedback monitor, and there were no significant differences in psychological distress between the early and late HRV provision groups at 12 months. This suggests that the HRV biofeedback component may not be essential for achieving the program’s primary objectives. This finding aligns with previous research that has questioned the efficacy of HRV biofeedback in certain populations [8].

Macdonald et al. (2024) reported that in the PC-PEP Phase 3 Clinical Trial, which compared a 6-month PC-PEP program with standard care, heart rhythm coherence outcomes did not show statistically significant changes following the intervention [7]. Moreover, heart rhythm coherence was not associated with the primary outcome of the study, psychological distress, at 6 months after the trial [7]. Similar findings were observed in a shorter version of the program, the 28-day PC-PEP, where heart rhythm coherence outcomes remained unchanged [42]. These results, combined with participants’ lower adherence to the HRV training and lower ratings in our study, suggest that participants may have derived greater benefits from other components of the intervention, such as physical activity, psychosocial tools, and dietary modifications. Streamlining CancerPEP by omitting the HRV component could improve the program’s integration into standard care practices while enhancing cost-effectiveness [7].

### 4.3. Implications for Cancer Survivorship Care

The findings of this study have important implications for cancer survivorship care. The positive outcomes associated with CancerPEP highlight the need for comprehensive, patient-centered interventions that address both the physical and psychological challenges faced by cancer survivors. As Murnaghan et al. [6] noted, many existing survivorship programs fail to adequately address the outcomes that matter most to patients, such as reintegration into daily life. CancerPEP’s multifaceted approach—encompassing physical, mental, and social support—represents a significant step forward in this regard. 

Moreover, the high levels of participant satisfaction and interest in continuing the program suggest that CancerPEP is well received by patients, further supporting its integration into routine cancer care. Sustained engagement is crucial for achieving long-term mental health and quality-of-life benefits, and CancerPEP’s structure facilitates such ongoing involvement.

### 4.4. Study Limitations and Future Research

While this study offers valuable insights into CancerPEP’s effectiveness, several limitations should be acknowledged. The study was conducted at a single site, potentially limiting the generalizability of the findings. Additionally, the sample was heterogeneous, including patients with various cancer types and at different stages of survivorship. Although this diversity provides a broad perspective, it precluded more detailed subgroup analyses, except for the breast cancer group, which was large enough for meaningful analysis. The variability in cancer types and stages may have introduced heterogeneity in the results, affecting the precision of the findings. However, the inclusion of patients with metastatic disease, who reported no adverse effects, suggests that CancerPEP could be safely extended to individuals with more advanced cancers.

A significant limitation of our study design is the absence of a true control group not receiving the CancerPEP intervention. This limitation has implications for interpreting the effectiveness of CancerPEP, as the results showing reductions in psychological distress may not definitively establish the program’s benefits without a formal comparison. Nonetheless, the observed declines in distress suggest the program’s potential efficacy in improving mental health outcomes among cancer patients.

The self-reported nature of distress assessments is another limitation, though the use of validated instruments such as the K10 mitigates this concern. Additionally, while this report focuses on outcomes up to 12 months, the study is ongoing, with 24-month evaluations planned to provide further insights into CancerPEP’s long-term effects.

A key strength of this study is the insight gained from the breast cancer sample, which has informed efforts to customize CancerPEP for breast cancer patients. This customization process will serve as a model for adapting the program to other cancer sites, allowing a more tailored approach to survivorship care. Importantly, a randomized clinical trial comparing the breast cancer group to recipients of usual care, similar to the approach taken in PC-PEP, is currently under way. This trial will include a health equity assessment to further explore the impact of CancerPEP on diverse populations.

Future research should replicate these findings across multiple sites and among a wider variety of cancer types to enhance generalizability. Additionally, exploring the long-term sustainability of the benefits observed, particularly beyond the 12-month mark, will provide further evidence of CancerPEP’s impact. Identifying which program components contribute most significantly to its success will also help refine the intervention for even greater efficiency and efficacy. The positive outcomes observed in patients with metastatic disease suggest that CancerPEP could be especially beneficial for individuals in more advanced stages of cancer.

## 5. Conclusions

In conclusion, the Cancer Patient Empowerment Program (CancerPEP) is an effective intervention for reducing psychological distress among cancer patients. The program’s holistic approach—incorporating physical activity, dietary guidance, and social support—effectively addresses the complex needs of cancer survivors, leading to significant improvements in mental health. Although the HRV biofeedback component did not enhance the program’s effectiveness, the overall success of CancerPEP highlights its potential as a valuable addition to survivorship care plans.

The positive outcomes observed across different cancer types, particularly in the breast cancer group, suggest that the PEP programs, including CancerPEP and PC-PEP, are broadly applicable across various cancers. The inclusion of patients with metastatic disease, without adverse effects, further supports the potential for expanding the program to individuals in more advanced stages of cancer.

As CancerPEP continues to be customized for breast cancer and other cancer types, it is poised to become a more tailored and versatile tool in survivorship care. Future research should focus on refining this intervention, ensuring its integration into standard cancer care worldwide. The ongoing 24-month evaluations will provide critical insights into the long-term sustainability of CancerPEP’s benefits.

## Figures and Tables

**Table 1:** The baseline characteristics of the 104 participating cancer patients residing in Canada are presented for the Cancer Patient Empowerment Program (CancerPEP) intervention groups: Early HRV (CancerPEP with the HRV biofeedback device) and Late HRV (CancerPEP without the HRV device initially, but with the option to use it for 6 months after the intervention).

	CancerPEP with Early HRV Device (*n* = 52)	CancerPEP with Late HRV Device (*n* = 52)	*p*-Value
Age (yr)	52, 60 (52–69)	52, 57 (51–61)	0.013
Sex ^1^ (female)	52, 45, 87%	52, 45, 87%	1.00
Menopause (menstrual period over one year ago)	37, 82%	28, 84%	0.8
Race	52, 49, 94%	52, 46, 89%	0.7
Sexual orientation, heterosexual	52, 52, 100%	52, 48, 92%	0.4
Body mass index	52, 28 (23–32)	52, 27 (23–30)	0.2
Household income, <$80,000 CAD/past year	52, 15, 29%	52, 11, 21%	0.4
Education, university or above	52, 34, 65%	55, 33, 64%	0.8
Employment status (full- or part-time)	52, 30, 58%	52, 27, 52%	0.6
Relationship status (married/currently in relationship)	52, 45, 87%	52, 43, 83%	0.6
Living			0.047
Rural	6, 12%	14, 27%	
Urban	46, 88%	38, 73%	
Province			0.6
Nova Scotia	9, 17%	8, 15%	
New Brunswick	0, 0%	2, 4%	
Newfoundland and Labrador	1, 2%	3, 6%	
Quebec	1, 2%	1, 2%	
Ontario	26, 50%	27, 52%	
Manitoba	0, 0%	1, 2%	
Saskatchewan	4, 8%	5, 10%	
Alberta	7, 13%	4, 7%	
British Columbia	4, 8%	1, 2%	
Screening positive for nonspecific psychological distress and need for clinical treatment (K10 ≥ 20)	52, 20, 39%	52, 24, 46%	0.4
Stage of cancer			0.5
I	14, 27%	10, 19%	
II or III	18, 35%	25, 48%	
IV	13, 25%	13, 25%	
Unknown	7, 13%	4, 8%	
Type of cancer			0.108
Bladder cancer	1, 2%	2, 4%	
Blood cancer	9, 17%	5, 10%	
Breast cancer	27, 52%	31, 59%	
Gynecological cancers (cervical, endometrial, ovarian, uterine)	4, 8%	3, 6%	
Colon or colorectal	6, 11%	1, 2%	
Kidney cancer	4, 8%	2, 4%	
Lung cancer	0, 0%	6, 11%	
Pancreatic cancer	0, 0%	1, 2%	
Skin cancer or melanoma	1, 2%	1, 2%	
Months between first cancer diagnosis and trial start	49, 27 (17, 58)	46, 30 (11, 34)	0.6
Metastasis present at trial start	3, 5.8%	3, 5.8	1.0
Treatment modality			0.7
Surgery ± radiation, hormone, and/or chemotherapy	41, 79%	39, 75%	
Radiation ± hormone (endocrine) therapy and/or chemotherapy	3, 6%	5, 10%	
Chemotherapy ± hormone (endocrine) therapy	8, 15%	8, 15%	
Multi-morbidities			0.6
One (other than the cancer diagnosis)	18, 35%	22, 42%	
Two	14, 27%	15, 29%	
Three or more	20, 38%	15, 29%	
Self-identified as a tobacco user	0, 0%	0, 0%	0.9
Currently using tobacco	0, 0%	0, 0%	
Used tobacco daily in the past	16, 30%	15, 28%	
Used tobacco less than daily in the past	4, 8%	3, 6%	
Never used tobacco	32, 62%	34, 66%	
Intake of prescribed medication for anxiety, depression, or both at the time of entry in the trial	9, 17%	12, 23%	

Summary statistics are presented as *n*, median, and interquartile range, or *n* (%) for categorical data. ^1^ Participants in the sample identified exclusively with two sex and gender categories—female and male. No other gender categories were reported.

**Table 2:** Multiple logistic regression analysis of nonspecific clinical psychological distress and need for treatment (K10 Score ≥ 20) at (A) 6-month follow-up (CancerPEP with vs. without biofeedback HRV monitor provided at trial start) and (B) at 12-month follow-up (CancerPEP with biofeedback HRV monitor provided at trial start vs. at 6 months after trial start), adjusted for prognostic covariates among 104 cancer patients in Canada.

**A.**	**Presence of psychological distress ** **and need for clinical treatment at 6 months ** **aOR (95% CI)**	***p***
Full cohort analysis ^a^ (*n* = 104)		<0.001
Group		
CancerPEP without biofeedback HRV monitor provided	1.0 Reference	
CancerPEP with biofeedback HRV monitor provided	0.72 (0.19, 2.69)	0.6
Psychological distress (K10) baseline	1.33 (1.15, 1.55)	<0.001
Partial cohort analysis ^a^ (n = 58; breast cancer patients only)		<0.001
Group		
CancerPEP without biofeedback HRV monitor provided	1.0 Reference	
CancerPEP with biofeedback HRV monitor provided	0.39 (0.05, 3.13)	0.4
Psychological distress (K10) baseline	1.54 (1.15, 2.08)	0.004
**B.**	**Presence of psychological distress ** **and need for clinical treatment at 12 months ** **aOR (95% CI)**	***p***
Full cohort analysis (*n* = 104)		0.002
Group		
CancerPEP with late (at 6 mo. after trial start) provision of biofeedback HRV monitor	1.0 Reference	
CancerPEP with early (at trial start) provision of biofeedback HRV monitor	1.14 (0.30, 4.39)	0.8
Psychological distress (K10) baseline	1.28 (1.11, 1.47)	<0.001
Partial cohort analysis (*n* = 58; breast cancer patients only)		0.010
Group		
CancerPEP with late (at 6 mo. after trial start) provision of biofeedback HRV monitor	1.0 Reference	
CancerPEP with early (at trial start) provision of biofeedback HRV monitor	0.24 (0.03, 1.98)	0.19
Psychological distress (K10) baseline	1.22 (1.02, 1.45)	0.028

Note: ^a^ Partial cohort analysis (breast cancer patients only); full cohort analyses models (A and B) are controlled for baseline K10 sum scores and include the following trial-entry prognostic covariates: patient’s age; treatment modality (surgery *±* radiation, hormone, and/or chemotherapy, radiation ± hormone and/or chemotherapy, or chemotherapy *±* hormone therapy); relationship status (not in a relationship vs. currently in a relationship); number of comorbidities (none, one, two, or three or more); prescribed medication for depression, anxiety, or both (yes vs. no); and days between the date of cancer diagnosis and the date of entry in the study.

**Table 3:** Generalized Estimating Equations (GEE) model results predicting psychological distress (K10 ≥ 20) across time points (trial start = Time 1, 6 months = Time 2, and 12 months = Time 3), adjusted for group randomization, and prognostic covariates among 104 cancer patients in Canada.

Variable	B	SE	95% CI	Wald χ²	df	*p*-Value	OR	95% CI for OR
Intercept	−3.26	1.36	[−5.92, −0.61]	5.79	1	0.016	0.04	[0.003, 0.55]
Time				18.13	2	<0.001		
Time 1 vs. Time 3	1.08	0.30	[0.49, 1.67]	12.72	1	<0.001	2.94	[1.62, 5.30]
Time 1 vs. Time 2	0.97	0.28	[0.42, 1.52]	12.14	1	<0.001	2.64	[1.53, 4.56]
Time 2 vs. Time 3	0.11	0.33	[−0.53, 0.75]	0.11	1	0.7	1.11	[0.59, 2.11]
Group randomization	0.57	0.38	[−0.16, 1.31]	2.33	1	0.13	1.77	[0.85, 3.70]
Patient age	−0.003	0.02	[−0.04, 0.04]	0.027	1	0.9	0.99	[0.96, 1.04]
Comorbidities	0.39	0.23	[−0.05, 0.83]	3.011	1	0.083	1.48	[0.95, 2.29]
Relationship status	0.16	0.49	[−0.80, 1.12]	0.11	1	0.7	1.17	[0.45, 3.07]
Treatment type	−0.06	0.25	[−0.56, 0.44]	0.057	1	0.8	0.94	[0.57, 1.55]
Prescribed intake of medication for anxiety, depression, or both	1.23	0.47	[0.31, 2.15]	6.88	1	0.009	3.42	[1.36, 8.55]
Months between diagnosis and enrollment	0.003	0.003	[−0.003, 0.01]	1.18	1	0.3	1.00	[0.99, 1.01]

Note: Time 3 serves as the reference category for time comparisons, and the first category serves as the reference for categorical variables. Exp(B) represents the odds ratio, which provides the magnitude of the effect of the predictors. Time 1: study start; Time 2: 6 months; Time 3: 12 months.

**Table 4:** Generalized Estimating Equations (GEE) model results predicting psychological distress (K10 ≥ 20) in the breast cancer patient cohort across time points (trial start = T1, 6 months = T2, and 12 months = T3), *n* = 58.

Variable	B	SE	95% CI	Wald χ²	df	*p*-Value	OR	95% CI for OR
Intercept	−4.73	2.39	[−9.41, −0.056]	3.93	1	0.047	0.01	[0.000082, 0.95]
Time				11.53	2	0.003		
Time 1 vs. Time 3	1.004	0.36	[0.300, 1.71]	7.82	1	0.005	2.73	[1.35, 5.52]
Time 1 vs. Time 2	0.81	0.39	[0.98,0.58]	7.06	1	0.008	2.25	[1.24, 4.08]
Time 2 vs. Time 3	0.19	0.39	[−0.58, 0.98]	0.24	1	0.6	1.22	[0.56, 2.65]
Group randomization	1.10	0.57	[−0.020, 2.22]	3.70	1	0.054	3.07	[0.98, 9.23]
Patient age	0.026	0.033	[−0.036, 0.088]	0.66	1	0.4	1.03	[0.96, 1.09]
Comorbidities	0.42	0.34	[−0.24, 1.08]	1.56	1	0.2	1.52	[0.79, 2.94]
Relationship status (currently in a relationship)	−0.020	0.69	[−1.37, 1.33]	0.001	1	0.9	0.98	[0.26, 3.78]
Treatment type	−0.60	0.57	[−1.73, 0.53]	1.093	1	0.3	0.55	[0.18, 1.69]
Prescribed intake of medication for anxiety, depression, or both	1.46	0.67	[0.152, 2.771]	4.78	1	0.029	4.31	[1.16, 15.98]
Months between diagnosis and enrollment	−0.000011	0.007	[−0.014, 0.014]	0.000	1	0.9	1.00	[0.99, 1.01]

Note: Time 3 serves as the reference category for time comparisons, and the first category serves as the reference for categorical variables. Exp(B) represents the odds ratio, which provides the magnitude of the effect of the predictors. Time 1: study start; Time 2: 6 months; Time 3: 12 months.

**Table 5:** Mixed-model analysis predicting psychological distress (K10 ≥ 20) among the full cancer patient sample, *n* = 104.

Variable	B	SE	Wald χ²	df	*p*	OR	95% CI
(Intercept)	−1.68	1.05	2.6	1	0.108	0.19	[0.024, 1.4]
Group (Late vs. Early HRV)	0.58	0.305	3.7	1	0.056	1.8	[0.99, 3.3]
Time 3 vs. Time 1	−1.08	0.35	9.4	1	0.002	0.34	[0.17, 0.68]
Time 2 vs. Time 1	−0.99	0.35	8.1	1	0.004	0.37	[0.19, 0.74]
Time 2 vs. Time 3	0.089	0.39	0.051	1	0.8	1.09	[0.52, 2.3]
Age	−0.001	0.015	0.003	1	0.9	0.99	[0.97, 1.03]
Comorbidities	0.36	0.17	4.2	1	0.041	1.4	[1.02, 2.004]
Relationship status (currently in a relationship)	0.16	0.43	0.14	1	0.7	1.2	[0.504, 2.7]
Prescribed intake of medication for anxiety, depression, or both	1.23	0.36	12	1	<0.001	3.4	[1.7, 6.9]
Months between diagnosis and enrollment	0.003	0.0028	1.4	1	0.2	1.003	[0.99, 1.009]
Treatment type	−0.062	0.21	0.092	1	0.8	0.94	[0.63,1.4]

**Table 6:** Mixed-model analysis predicting psychological distress (K10 ≥ 20) in the breast cancer Subgroup, *n* = 58.

Variable	B	SE	Wald χ²	df	*p*	OR	95% CI
(Intercept)	−2.6	1.6	2.6	1	0.104	0.073	[0.003, 1.7]
Group (Late vs. Early HRV)	1.1	0.43	6.9	1	0.009	3.11	[1.3, 7.2]
Time 3 vs. Time 1	−1.02	0.47	4.6	1	0.031	0.36	[0.14, 0.91]
Time 2 vs. Time 1	−0.87	0.47	3.5	1	0.062	0.42	[0.17, 1.05]
Time 2 vs. Time 3	0.15	0.51	0.086	1	0.8	1.2	[0.43, 3.1]
Age	0.028	0.022	1.7	1	0.19	1.03	[0.99, 1.07]
Comorbidities	0.39	0.23	2.8	1	0.096	1.48	[0.93, 2.3]
Relationship status (currently in a relationship)	0.015	0.59	0.001	1	0.98	1.02	[0.32, 3.3]
Prescribed intake of medication for anxiety, depression, or both	1.5	0.49	9.1	1	0.003	4.5	[1.69, 12]
Months between diagnosis and enrollment	0.000	0.0052	0.008	1	0.93	1.0	[0.99, 1.01]
Treatment type	−0.74	0.59	1.8	1	0.209	1.7	[0.15, 1.5]

**Table 7:** Participant exit evaluations at 6 months upon completion of the CancerPEP program (*n* = 96).

	CancerPEP Intervention with HRV Device, *n* = 49 Responses Ranged from 0 (Not at All) to 10 (Extremely)			CancerPEP Intervention without HRV Device, *n* = 47 Responses Ranged from 0 (Not at All) to 10 (Extremely)		
	*n*	Mean	SD	*n*	Mean	SD
Participant-perceived competence of the CancerPEP Research and Clinical Team	49	9.39	1.06	47	9.13	1.28
Participant-rated likelihood of recommending the CancerPEP program to others who have been diagnosed with cancer	49	9.41	1.22	47	9.17	1.55
Participant-perceived importance of implementing CancerPEP as part of the standard of care for patients diagnosed with cancer, from the day of diagnosis	49	9.00	1.53	47	9.00	1.55
Participant’s interest in the CancerPEP program after first learning about it	49	8.96	1.41	47	9.17	1.22
Participant-perceived usefulness of the introduction/training videos at baseline	49	8.33	1.74	47	8.09	1.73
Participant-perceived overall usefulness of the CancerPEP program	49	8.57	1.56	47	8.11	2.15
Participant-perceived accessibility and quick response to inquiries throughout the trial from the CancerPEP research team and staff	49	8.63	2.05	47	8.26	2.13
Participant-perceived usefulness of the program’s strength and aerobic exercises	49	7.20	2.15	47	7.40	2.64
Participant-perceived usefulness of the program’s yoga videos and exercises (optional)	21	6.81	2.46	32	7.31	2.52
Participant-perceived usefulness of the program’s dietary/nutrition advice and materials	49	8.33	1.70	47	8.13	2.46
Participant’s perceived lifestyle benefits at the end of 6 months compared to the start of the program (baseline)	49	7.73	1.41	47	7.55	1.98
Participant-perceived usefulness of the program’s website, which included resources and program information	49	7.94	2.08	47	7.74	2.35
Participant-perceived usefulness of the program to their partner (if applicable) during the 6 months	49	6.41	3.39	47	6.43	3.54
Participant-perceived usefulness of the program’s daily videos with education and empowerment messages	49	8.65	1.47	47	8.34	2.23
Participant-perceived usefulness of the program’s intimacy and connection education materials (videos and daily messages)	49	7.04	2.14	47	7.15	2.81
Participant-perceived usefulness of the program’s healthy sleep habits component	49	7.45	1.84	47	7.32	2.76
Participant-perceived usefulness of the program’s bi-weekly videoconference (if attended)	39	7.15	2.55	33	7.42	2.46
Participant-perceived usefulness of the program’s stress reduction biofeedback device (HRV monitor)	49	4.88	3.52	*n*/a	n/a	n/a
Participant-perceived usefulness of the program’s meditation videos	49	7.37	2.44	47	7.49	2.75
Participant-perceived usefulness of the program’s Saturday weekly educational videos	49	6.43	2.87	47	6.45	3.06
Participant-perceived usefulness of learning about habit formation and breaking bad habits	49	7.98	1.51	47	7.32	2.89
Participant-perceived usefulness of the teachings from the book Love is Letting Go of Fear	48	6.42	2.70	46	6.50	3.16
Participant-perceived usefulness of the Facebook group	39	6.79	1.91	34	7.53	2.44
Participant-perceived usefulness of the program’s Program Partner system (connection with 2 co-participants attending and going through the program at the same time and facing a similar cancer diagnosis and/or treatment) (optional program component)	23	6.52	3.10	30	6.23	3.14
Interest in continuing with the program after the 6 months	49	30	61%	47	36	77%
Participants’ interest in becoming a Mentor for the CancerPEP program	49	11	22%	47	16	34%
Participants’ interest in becoming a Research Citizen for the CancerPEP program	49	24	49%	47	15	32%

Note: SD = standard deviation; HRV = heart rate variability; CancerPEP = Cancer Patient Empowerment Program.

## Data Availability

Data from this study are available to researchers through a data access process in compliance with patient privacy and protection research acts (NSHA Research Ethics Board).

## Supplementary Materials

The following supporting information can be downloaded at: https://www.mdpi.com/article/10.3390/cancers16193373/s1, S1. CONSORT 2010 checklist of information to include when reporting a randomized trial; S2. Study protocol; S3. Table: Results of Generalized Estimating Equations analysis for psychological distress (K10 ≥ 20) in the complete study cohort, *n* = 104, with the group-by-time interaction term; S4. Table: Results of Generalized Estimating Equations analysis for psychological distress (K10 ≥ 20) in the breast cancer cohort with the group-by-time interaction term, n = 58; S5 Table: Tests of model effects for mixed-model analysis in the full study cohort, n = 104, with the group-by-time interaction term; S6 Table: Tests of model effects for mixed-model analysis in the breast cancer subsample, n = 58, with the group-by-time interaction term.
