# Supplementary material for: The Cancer Patient Empowerment Program: A Comprehensive Approach to Reducing Psychological Distress in Cancer Survivors, with Insights from a Mixed-Model Analysis, Including Implications for Breast Cancer Patients
Source: Cancers (Basel). 2024 Oct 2;16(19):3373. doi: 10.3390/cancers16193373 (PMC11475673; doi:10.3390/cancers16193373)
Supplement: Supplementary file 1 [file cancers-16-03373-s001.zip › cancers-3226156-supplementary.docx]

# **Supplementary Material (*Ilie et al., 2024, Cancers)***

# *S1.CONSORT*


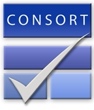
CONSORT 2010 checklist of information to include when reporting a randomised trial*

| Section/Topic | Item No | Checklist item | Reported on page No |
| --- | --- | --- | --- |
| Title and abstract | | | |
|  | 1a | Identification as a randomised trial in the title | 1 |
|  | 1b | Structured summary of trial design, methods, results, and conclusions (for specific guidance see CONSORT for abstracts) | 1-2 |
| Introduction | | | |
| Background and objectives | 2a | Scientific background and explanation of rationale | 2-4 |
|  | 2b | Specific objectives or hypotheses | 3-4 |
| Methods | | | |
| Trial design | 3a | Description of trial design (such as parallel, factorial) including allocation ratio | 4-5 |
|  | 3b | Important changes to methods after trial commencement (such as eligibility criteria), with reasons | N/A |
| Participants | 4a | Eligibility criteria for participants | 4-5 |
|  | 4b | Settings and locations where the data were collected | 4 |
| Interventions | 5 | The interventions for each group with sufficient details to allow replication, including how and when they were actually administered | 4-6 |
| Outcomes | 6a | Completely defined pre-specified primary and secondary outcome measures, including how and when they were assessed | 5-7 |
|  | 6b | Any changes to trial outcomes after the trial commenced, with reasons | N/A |
| Sample size | 7a | How sample size was determined | 7 |
|  | 7b | When applicable, explanation of any interim analyses and stopping guidelines | N/A |
| Randomisation: |  |  | 5 |
| Sequence generation | 8a | Method used to generate the random allocation sequence |  |
|  | 8b | Type of randomisation; details of any restriction (such as blocking and block size) | 5 |
| Allocation concealment mechanism | 9 | Mechanism used to implement the random allocation sequence (such as sequentially numbered containers), describing any steps taken to conceal the sequence until interventions were assigned | 4 |
| Implementation | 10 | Who generated the random allocation sequence, who enrolled participants, and who assigned participants to interventions | 4 |
| Blinding | 11a | If done, who was blinded after assignment to interventions (for example, participants, care providers, those assessing outcomes) and how | 4 |
|  | 11b | If relevant, description of the similarity of interventions | N/A |
| Statistical methods | 12a | Statistical methods used to compare groups for primary and secondary outcomes | 7-8 |
|  | 12b | Methods for additional analyses, such as subgroup analyses and adjusted analyses | 7-8 |
| Results | | | |
| Participant flow (a diagram is strongly recommended) | 13a | For each group, the numbers of participants who were randomly assigned, received intended treatment, and were analysed for the primary outcome | 5 |
|  | 13b | For each group, losses and exclusions after randomisation, together with reasons | 9 |
| Recruitment | 14a | Dates defining the periods of recruitment and follow-up | 4 |
|  | 14b | Why the trial ended or was stopped | N/A |
| Baseline data | 15 | A table showing baseline demographic and clinical characteristics for each group | 8-9 |
| Numbers analysed | 16 | For each group, number of participants (denominator) included in each analysis and whether the analysis was by original assigned groups | 5,8 |
| Outcomes and estimation | 17a | For each primary and secondary outcome, results for each group, and the estimated effect size and its precision (such as 95% confidence interval) | 11, 13-14 |
|  | 17b | For binary outcomes, presentation of both absolute and relative effect sizes is recommended | 11, 13-14 |
| Ancillary analyses | 18 | Results of any other analyses performed, including subgroup analyses and adjusted analyses, distinguishing pre-specified from exploratory | 13-14 |
| Harms | 19 | All important harms or unintended effects in each group (for specific guidance see CONSORT for harms) | N/A |
| Discussion | | | |
| Limitations | 20 | Trial limitations, addressing sources of potential bias, imprecision, and, if relevant, multiplicity of analyses | 18 |
| Generalisability | 21 | Generalisability (external validity, applicability) of the trial findings | 18 |
| Interpretation | 22 | Interpretation consistent with results, balancing benefits and harms, and considering other relevant evidence | 16-18 |
| Other information | | |  |
| Registration | 23 | Registration number and name of trial registry | 4 |
| Protocol | 24 | Where the full trial protocol can be accessed, if available | Supplementary Material |
| Funding | 25 | Sources of funding and other support (such as supply of drugs), role of funders | 19 |

# *S2. Study Protocol*

**CANCER–PEP PHASE 2 TRIAL PROTOCOL**

**CancerPEP Phase 2 Randomized Trial: Feasibility of a Comprehensive Cancer Patient Empowerment Program providing immediate versus delayed stress reduction biofeedback equipment**

**Principal Investigator:** Dr. Gabriela Ilie

**Coordinating Centre:** QEII Health Sciences Centre, Halifax, NS

**Funded by:** Dalhousie Medical Research Foundation

**Version Number:** Version 2

**Version Date:** November 23, 2022

**Prepared by:** Dr. Gabriela Ilie, Dr. Rob Rutledge, and Cody MacDonald

**Document #:** CANCER_PEP_Phase 2_Protocol_Version_2_Nov_23 _2022

**CONFIDENTIALITY STATEMENT**

This document is confidential communication. Acceptance of this document constitutes agreement by the recipient that no unpublished information contained herein will be published or disclosed without prior approval of the Principal Investigator or other participating study leadership.

**TABLE OF CONTENTS**

[STATEMENT OF COMPLIANCE 4](#_Toc119942852)

[INVESTIGATOR’S SIGNATURE 4](#_Toc119942853)

[1 PROTOCOL SUMMARY 5](#_Toc119942854)

[**1.1 SYNOPSIS** 5](#_Toc119942855)

[**1.2 SCHEMA** 7](#_Toc119942856)

[2 BACKGROUND 8](#_Toc119942857)

[**2.1 Rationale for the Cancer Patient Empowerment Program** 8](#_Toc119942858)

[**2.2 Scientific Basis for a Patient Empowerment Program** 8](#_Toc119942859)

[2.2.1 Aerobic exercise 8](#_Toc119942860)

[2.2.2 Strength Training 8](#_Toc119942861)

[2.2.3 Yoga 9](#_Toc119942862)

[2.2.4 Safety of aerobic and strength training for cancer patients 9](#_Toc119942863)

[2.2.5 Stress Reduction through Meditation and Relaxation Exercises 10](#_Toc119942864)

[2.2.6 Dietary Advice 10](#_Toc119942865)

[2.2.7 Relationship training 11](#_Toc119942866)

[2.2.8 Building on Pre-habilitation (Prior to Treatment) Science 11](#_Toc119942867)

[**2.3 Comprehensive Programming vs Individual Element Health Promotion** 12](#_Toc119942868)

[**2.4 Brief Description of the Cancer Patient Empowerment Program (CancerPEP)** 13](#_Toc119942869)

[**2.5 Prostate Cancer PEP Feasibility Trial Results** 13](#_Toc119942870)

[**2.6 Prostate Cancer PEP Phase 3 Trial Results** 14](#_Toc119942871)

[**2.7 Prostate Cancer PEP Phase 4 Trial** 15](#_Toc119942872)

[**2.8 Difference between PC-PEP Phase 4 and this CancerPEP Phase 2 Proposal** 15](#_Toc119942873)

[2.8.1 Random Assignment of ‘Early’ vs ‘Late’ Access to Heart Rate Variability Monitors 15](#_Toc119942874)

[**2.9 Purpose of the study** 16](#_Toc119942875)

[3 STUDY OBJECTIVES AND OUTCOMES 16](#_Toc119942876)

[**3.1 Primary Objective** 16](#_Toc119942877)

[**3.2 Secondary Objectives** 17](#_Toc119942878)

[4 SELECTION AND ENROLLMENT OF PARTICIPANTS 18](#_Toc119942879)

[**4.1 Eligibility Criteria** 18](#_Toc119942880)

[4.1.1 Inclusion Criteria 18](#_Toc119942881)

[4.1.2 Exclusion Criteria 18](#_Toc119942882)

[**4.2 Recruitment, Screening, and Randomization** 19](#_Toc119942883)

[4.2.1 Recruitment Methods and Timeline 19](#_Toc119942884)

[4.2.2 Pre-screening 19](#_Toc119942885)

[4.2.3 Informed Consent 20](#_Toc119942886)

[4.2.4 Baseline Survey 21](#_Toc119942887)

[4.2.5 Randomization 21](#_Toc119942888)

[5 CLINICAL INTERVENTION (Cancer Patient Empowerment Program) 21](#_Toc119942889)

[**5.1 Interventions and Administration** 21](#_Toc119942890)

[5.1.1 Aerobic Training Recommendations 22](#_Toc119942891)

[5.1.2 Strength Training and Yoga Recommendations 22](#_Toc119942892)

[5.1.3 Logistics of Strength Training and Yoga program 23](#_Toc119942893)

[5.1.4 Relaxation Technique Instruction 23](#_Toc119942894)

[5.1.5 Logistics of Heart Rate Variability (HRV) Monitors and Training 24](#_Toc119942895)

[5.1.6 Healthy Lifestyle Promotion 24](#_Toc119942896)

[5.1.7 Smoking Cessation 24](#_Toc119942897)

[5.1.8 Connection and Relationship Teaching 25](#_Toc119942898)

[5.1.9 Social Support 25](#_Toc119942899)

[5.1.10 Weekly Schedule 25](#_Toc119942900)

[5.1.11 Weekly Compliance Survey 26](#_Toc119942901)

[5.1.12 Biweekly Videoconferencing with active PEP participants 26](#_Toc119942902)

[5.1.13 Using Technology for Support 27](#_Toc119942903)

[5.1.14 Bank of Broadcasted Emails and Videos 27](#_Toc119942904)

[6 WITHDRAWAL OF PARTICIPANTS 28](#_Toc119942905)

[**6.1 Protocol Violation** 28](#_Toc119942906)

[**6.2 Withdrawal of Participants** 28](#_Toc119942907)

[7 ADVERSE EVENTS AND REPORTING REQUIREMENTS 28](#_Toc119942908)

[**7.1 Adverse Events** 28](#_Toc119942909)

[**7.2 Indications for Stopping Exercise** 29](#_Toc119942910)

[**7.3 Reporting Requirements** 29](#_Toc119942911)

[8 CLINICAL EVALUATIONS 30](#_Toc119942912)

[**8.1 Schedule of Evaluations** 30](#_Toc119942913)

[**8.2 Online Questionnaires** 30](#_Toc119942914)

[9 STATISTICAL CONSIDERATIONS 32](#_Toc119942915)

[**9.2 Analysis of Primary Outcome** 32](#_Toc119942916)

[**9.3 Analysis of Secondary Quantitative Outcomes** 33](#_Toc119942917)

[**9.4 Analysis of Qualitative Outcomes** 33](#_Toc119942918)

[10 PROTECTION OF HUMAN SUBJECTS 34](#_Toc119942919)

[**10.1 Confidentiality** 34](#_Toc119942920)

[**10.2 Harms** 34](#_Toc119942921)

[**10.3 Benefits** 35](#_Toc119942922)

[**10.4 Liability** 35](#_Toc119942923)

[**10.5 Disclosure of Any Financial Compensation** 35](#_Toc119942924)

[11 PUBLICATION OF RESEARCH FINDINGS 35](#_Toc119942925)

[**11.1 Dissemination Plan** 35](#_Toc119942926)

[LIST OF ABBREVIATIONS 36](#_Toc119942927)

#

# **STATEMENT OF COMPLIANCE**

The trial will be conducted in accordance with this protocol, International Council on Harmonisation Good Clinical Practice (ICH GCP) and applicable regulatory requirements. The Principal Investigator (PI) will assure that no deviation from, or changes to the protocol will take place without an amendment and documented approval from the Research Ethics Board (REB), except where necessary to eliminate (an)immediate hazard(s) to the trial participants.

The protocol, informed consent form(s), recruitment materials, and all participant materials will be submitted to the REB for review and approval. Approval of both the protocol and the consent form(s) must be obtained before any participant is enrolled. Any amendment to the protocol will require review and approval by the REB before the changes are implemented to the study. All changes to the consent form will be REB approved; a determination will be made regarding whether a new consent needs to be obtained from participants who provided consent, using a previously approved consent form.

# **1 PROTOCOL SUMMARY**

## **1.1 SYNOPSIS**

| Title | CancerPEP Phase 2 Randomized Trial: Feasibility of a Comprehensive Cancer Patient Empowerment Program providing immediate versus delayed stress reduction biofeedback equipment |
| --- | --- |
| Funder(s) | Dalhousie Medical Research Foundation |
| Primary Objective | To determine the feasibility and participants’ safety during the offering of a six-month comprehensive home-based cancer patient empowerment (Cancer PEP) program for all cancer types |
| Secondary Objectives | 1. To determine whether providing stress reduction biofeedback equipment during the program changes mental health outcomes and participants’ experience 2. To assess patient satisfaction with the program including obtaining feedback on all components of the program 3. To determine the demographic, cancer type/stage, psychological, social, medical and treatment-related risk factors predicting poor mental health and quality of life at the end of the six-month program and 6 and 18 months later |
| Number of participants | 100 |

| Enrollment Criteria | -Participants must have a history of cancer of any type or stage  -Predicted life expectancy of greater than 1 year  -Deemed safe to participate in a low to moderate intensity exercise program including light strength training  -Able to read English and access daily emails, YouTube videos and Zoom for six months  -Older than 18 years of age |
| --- | --- |
| Primary Endpoints | Feasibility of the comprehensive program will be measured by accrual rate, attrition rate and weekly compliance to program activities. Safety will be assessed by monthly online questionnaires and by self-report. |
|  |  |
| Other Evaluations | Mental health at six months will be measured by the Kessler Psychological Distress Scale (K10) to test the effect of a heart-rate variability (HRV) biofeedback monitor during the six months of the program. Qualitative interviews will also assess the experience of the participants randomly assigned to immediate versus delayed access to HRV monitors  In addition, all participants will complete online surveys at baseline, and at 6, 12 and 24 months (where possible) including  the Kessler Psychological Distress Scale, Dyadic Assessment Scale, Screening for Distress Questionnaire, Quality of life (SF-12 and FACT-G), Perceived Illness Questionnaire, Structural-Functional Social Support Scale, The Pittsburgh Sleep Quality Index, The Sedentary Behaviour Questionnaire, and   1. Self-reported cancer type, stage, survivorship time since diagnosis, and treatment 2. Baseline demographic and personality assessment 3. Exit quantitative assessments including patient satisfaction 4. Exit qualitative interviews |
| Rationale for number of patients | A sample of 100 Canadian cancer patients will provide the feedback to address the primary outcome of feasibility and safety |
| Analysis Plan for Study Endpoints | The primary endpoint (feasibility) involves simple report of accrual, attrition and compliance rates, and safety by number of self-reported and otherwise discovered adverse events at 6 months.  Mental health (K10 questionnaire) will be compared between the immediate vs delayed access to the biofeedback monitor conditions at the six month point by an independent t test evaluating different scores of total psychological distress from baseline to 6 months.  Participants’ mental health outcomes at 6 months will be compared against a sample of 200+ prostate cancer patients who have completed a similar 6-month program (PC-PEP) through an independent t test to see if, overall, there are differences in the mental health outcome due to cohort testing (prostate versus all other types of cancer). |
| Study Duration: | The study will be completed in approximately three years from first enrolment to the completion of data collection. |
| Participant Duration: | Participants will take two years to complete all study-related tasks, including six months of a home-based program and four online surveys at baseline, 6, 12 and 24 months. |

## **1.2 SCHEMA**

Prior to

Participant pre-screens themselves for eligibility,

or speaks to RC for pre-screening

Enrollment

RC fully explains study and obtains informed consent

Participant completes baseline questionnaire

Baseline

Randomize

Administer study intervention with HRV monitor. Monthly safety assessment.

Administer the study intervention without HRV monitor. Monthly safety assessment.

Day 1 -182

6- month questionnaire, exit survey, and (optional) end -of-intervention interview/focus

Arm 2 receives HRV monitor for 6 months

Day 182

12-month questionnaire

Day 365

24-month questionnaire

Day 730

Study Analyses

Analysis

# **2 BACKGROUND**

## **2.1 Rationale for the Cancer Patient Empowerment Program**

Each year over 230,000 Canadians are diagnosed with cancer. An overall 5-year survival rate of 64% has resulted in over 1 million cancer survivors living in Canada at any time ^1^. A cancer diagnosis can have profound effects on a person’s life - functional, emotional, social, and financial. Mental health issues are prevalent in cancer survivors with at least a third of patients expected to suffer from significant anxiety or depression during their journey ^2–4^. The current epidemic of loneliness and disconnect extends to people with a history of cancer, further worsening the adverse psychological consequences of the diagnosis ^5–7^. Cancer survivors are also at increased risk of chronic medical conditions like cardiovascular disease, obesity, diabetes and many others ^8,9^. Overall, a cancer diagnosis can have a profound impact on survivors’ mental and physical health in the short and long term. Few multifaceted behavioural interventions exist that engage cancer patients and survivors on a daily basis and target the areas affecting cancer patients’ lives to the extent of what is proposed in CancerPEP.

## **2.2 Scientific Basis for a Patient Empowerment Program**

Promotion of healthy lifestyle habits in cancer survivors has been extensively studied and was shown to improve quality of life, and, in some cancers, influenced disease progression. Each of the health-promoting activities incorporated in this study (CancerPEP) is based on strong scientific evidence.

### 2.2.1 Aerobic exercise

Multiple studies of exercise interventions for cancer patients have identified physical activity as an effective means of improving both physiological and psychological health following a diagnosis ^10–12^. In a review of over 30 studies and several meta-analyses of 1844 patients examining the effects of physical activity through exercise interventions during and after cancer treatment, it was shown that physical activity resulted in improved cardiorespiratory fitness, muscle strength, flexibility, depression, pain, anxiety, fatigue and quality of life in cancer patients of different types and stages of cancers both during and after treatment ^13^. Further, in a recent systematic review that included hundreds of epidemiologic studies with several million participants, both high and low levels of physical activity were shown to lower the risks and improve survival in cancer patients of breast, colon, bladder, endometrial, esophageal, gastric, and renal cancers and reduce mortality for breast, colon, and prostate cancer survivors ^14^.

### 2.2.2 Strength Training

Improved strength has been associated with improved daily performance, overall functionality, quality of life and reduced risk of falls ^15^. Cancer treatments have been associated with unfavorable changes in patients’ quality of life, body composition, physical function, and strength ^15^. Benefits of strength training vary in different cancer types but one consistent measure that improved was strength in patients with breast, prostate, lung, neck and head, lymphoma, colorectal and gastro-intestinal cancers ^15,16^. In Canada, a randomized controlled trial using 242 breast cancer patients evaluating the effect of strength training on quality of life has shown that it reduced anxiety and depression and improved fatigue and quality of life in breast cancer patients undergoing chemotherapy ^17^. Similarly, in a 14-week exercise intervention in 20 head and neck cancer patients investigating the effect of exercise showed maintained or improved quality of life and function in cancer patients undergoing chemotherapy ^18^. Both systematic reviews and meta-analyses of over 60 publications have shown that strength training had a positive effect and improved muscle functioning, body composition, oxygen-uptake and quality of life in cancer survivors ^19–22^.

### 2.2.3 Yoga

Cancer has been reported to influence different aspects of patients’ life and many quality-of-life domains were correlated with different mental health domains in cancer patients ^23^. Practicing yoga as a gentle form of physical activity has potential benefit in managing psychological distress, depression, anxiety, and stress and improving quality of life in many cancer survivors that they face due to their cancer diagnosis ^24^. Multiple studies in breast cancer patients have shown that yoga improved reported psychological distress, coordination, sleep, and digestion and reduced reported depression, pain, fatigue, and stress ^25–27^. Further, an analysis of 25 yoga interventions conducted with cancer survivors has shown evidence in 13 interventions for the clinical significance of yoga for improving cancer survivors’ psychosocial measures in the areas of depression, anxiety, and spiritual well-being, symptoms like fatigue, sleep, and overall quality of life ^28^.

### 2.2.4 Safety of aerobic and strength training for cancer patients

Aerobic and resistance exercise programs are safe and feasible for cancer patients to undertake during treatment ^11,29^. The push for exercise as an aid to cancer patients in combatting treatment effects is due to the potential that exercise can improve potency and delivery of treatment while enhancing the patients’ ability to tolerate the intervention ^30^. Exercise may also suppress tumor formation and further metastases ^30^. Cancer patients with bone metastases are recommended to avoid exercises that directly load weight to skeletal regions where the metastatic legions exist in order to avoid injury ^31^. There are concerns that resistance training may not be as safe for advanced cancer patients, therefore in these cases aerobic training may be more suitable ^32^. As well, the program may be autoregulated to fit the progression and current needs of the patient ^30^. However, in a recent systematic review of 14 exercise interventions assessing the safety, feasibility, and benefits of exercise for patients with advanced cancer, it has been concluded that strength and aerobic exercises were feasible, safe, and may lower symptoms like chronic fatigue in cancer patients with two studies showing significant improvements in cancer patients’ quality of life post intervention ^33^.

#### **2.2.4.1 Exercise Recommendations for Participants with Advanced Cancer**

According to research, aerobic exercise such as moderate to high intensity movements that provide stress to the cardiorespiratory system are recommended for advanced prostate cancer patients and those with bone metastases ^30^. Other workout modalities such as resistance exercise and moderate intensity continuous training are also recommended with caution. Impact exercise is not recommended for patients with bone metastases as it is not as tolerated and may increase risk of fracture, but patients without bone metastases can perform impact exercise in moderation ^30^. Participants with known metastases should also be monitored closely during exercise and should avoid any weight bearing exercises applying torsion to the bone as to avoid fracture. As well, high-intensity interval training is recommended but with caution as there is an increased fall risk post exercise ^30^. Specific exercise intolerances in prostate cancer survivors may be contributed to location of inoperable tumours, reiterating the importance of an individualistic exercise routine for each patient ^34^. This protocol complies with all these recommendations.

### 2.2.5 Stress Reduction through Meditation and Relaxation Exercises

Participating in a meditation or mindfulness program has been consistently reported to reduce stress symptoms ^35–40^, mood disturbance ^35,36,41^ ,anger ^39^, and fatigue ^38^ as well as improve quality of sleep ^40^, facilitate post-traumatic growth ^38^, promote spirituality ^38,42^ and enhance quality of life ^41^ in survivors of various types of cancer. Men with prostate cancer who are under medical surveillance reported significantly greater resilience and less anxiety after receiving an intervention of mindfulness meditation ^41^.

The implementation of meditation biofeedback training using heart rate variability (HRV) has been shown to improve the quality of life in study participants experiencing many conditions such as diabetes ^43^, hypertension ^44^, asthma ^45,46^, PTSD ^47^, chronic pain ^48^, and prostate cancer ^49,50^. HRV biofeedback training can decrease emotional and physiological stress levels ^51^, symptoms of burnout, anxiety, and depression ^51–53^ and increase working and episodic memory ^54^ and emotional well-being ^47,55,56^. In the pilot intervention of PC-PEP with 30 prostate cancer survivors over the 28-day program, participation resulted in a significant decline in reported psychological distress to 7% post intervention from 27% pre intervention and participants’ average mediation was 66.23 minutes per week ^49^. It was concluded that the PC-PEP program has the potential to positively impact the mental health and well-being of prostate cancer survivors at different stages ^49^. Similar results were found in phases three and four of PC-PEP which suggests the impact that meditation can have on survivors’ mental health and improved well-being.

### 2.2.6 Dietary Advice

Scientific evidence has reported the promising potential of healthy nutrition being the most preventive factor of cardiovascular death and even reversing heart disease ^57,58^. Breast cancer patients and patients with chronic illnesses (e.g., rheumatoid arthritis) have expressed interest in changing dietary habits, leaning towards healthier diets and the need for precise recommendations for individual disease situations ^59^. In a systematic review of twelve studies with different cancer patients, health-related quality of life outcomes were shown to improve as a result of dietary changes in cancer survivors, with prostate, breast and colorectal cancer survivors benefiting the most ^60^. Dietary advice was shown to be effective for improving treatment-related symptoms, dietary intake, and quality of life in cancer patients during treatment ^61–63^. In addition, a recent cohort study of 6655 prostate cancer patients has shown an association between a higher consumption of plant-based diet and reduced risk of advanced, lethal, and fatal prostate cancer ^63^. Similarly, in a randomized controlled trial assessing the effects of diet and physical activity interventions in 223 adult Chinese colorectal cancer survivors on anxiety, depression, and quality of life, it was shown that patients receiving the dietary intervention experienced improvements in their quality of life and levels of depression for up to two years post intervention ^64^.

### 2.2.7 Relationship training

Relationship training is essential in the patient’s cancer journey as it has been suggested that breast and gynecological cancer patients who received a couple-skills intervention reported less fear of their cancer prognosis, less avoidance in dealing with the cancer and better relationship skills ^65^. Similarly, psychosocial interventions that provided relationship training showed improved depression and quality of life in prostate cancer patients ^66^. A study of Chinese patients with breast cancer investigating the role of social support on resilience and quality of life found that patients with strong social support experienced improved resilience and quality of life ^67^. Further, in a systematic review investigating the effect of 23 psychosocial interventions on psychological distress (e.g., anxiety, depression) and quality of life in cancer patients of different types found that couples-based interventions that focused on improving partners communication skills resulted in improved quality of life and psychological distress like anxiety, depression, worry, negative thoughts or mood ^68^.

### 2.2.8 Building on Pre-habilitation (Prior to Treatment) Science

Participating in preoperative exercise programs, and/or having a higher level of health and fitness immediately prior to surgery, appears to facilitate a quicker recovery and fewer complications in patients undergoing many operations ^69–72^. In an Australian feasibility study (n=10) that provided a 6-week program of both resistance and aerobic exercises prior to surgery to recently diagnosed men (mean age = 68) with localized prostate cancer, the results showed improved physical performance and muscle strength with physical performance benefits lasting 6 weeks after surgery, and no adverse effects were reported ^73^. Additionally, a study in the Netherlands conducted on 70 breast cancer patients of different stages who were 75 years or younger evaluating the psychological effects of stress management training prior to surgery found reduced depression and fatigue post-surgery in patients who received the training compared to patients who did not ^74^. Similarly, in a North American study assessing the immune outcomes of stress management before surgery in 159 adult prostate cancer patients using blood samples found that immune parameters were increased post-surgery and reduced pre-surgical mood-disturbances ^75^. In a recent Chinese randomized controlled trial conducted with 73 lung cancer patients, a 2-week multimodal intervention program pre-surgery including nutritional counseling, mental relaxation, exercise, and respiratory training showed greater functional capacity through an increased pre-operative 6-minute walk test distance (+41.3m) compared with the control group ^76^.

## **2.3 Comprehensive Programming vs Individual Element Health Promotion**

Research has shown that multicomponent programs target different patient needs, symptoms or side effects at the same time and allow them to improve their quality of life ^77^. In a multifaceted intervention, the effects of group-mediated cognitive behavioral exercise and diet intervention was compared to standard of care on certain social cognitive outcomes, and it has shown improved outcomes in prostate cancer patients ^78^. Multiple trials have tested programs with single elements (exercise, or mindfulness /meditation, or social support) ^56,79,80^ and less so with two elements (e.g., exercise plus social support) ^81^. However, the cancer patient empowerment program is the first intervention we know of which combines multiple elements into a daily home-based online program (see below for details)

Patient activation highlights the importance of patients’ motivation, understanding their role and their willingness to take independent actions to be in charge of their own health and care ^82^. Multiple studies have shown that more activated patients are two or more times more likely than less activated patients to have more knowledge about their condition’s treatment guidelines, look for more health information, and prepare better for their doctor’s visit ^83,84^, thus improving their health outcomes and care experiences ^82^. Similarly, in a study including over 500 cancer survivors of different cancers has shown that highly activated patients are overall more informed about their cancer and treatment plan, understanding it and proactively cope with their symptoms/ side effects ^85^. In a recent meta-analysis of 26 interventions in Chinese or English on adults with chronic illness like cardiovascular disease, cancer, chronic respiratory disease, or diabetes, it was concluded that interventions that encouraged patient activations showed significant improvements in patients’ physiological, psychosocial like depression and anxiety and health-related quality of life ^86^. Patient empowerment is important as the need for cancer patients to understand and process their cancer diagnosis related information and instructions by their healthcare providers is crucial to be able to make informed decisions for their treatment options, understand the oncological care system better and improve their health outcomes ^87,88^. A study that included 264 breast cancer survivors has shown that patient empowerment had significant positive influence on their quality of life ^89^. Further, in a study on haematological cancer patients (n=7), it was highlighted that patient empowerment improved quality of life, resulted in positive mental attitude, and better coping with treatment and care ^90^.

## **2.4 Brief Description of the Cancer Patient Empowerment Program (CancerPEP)**

See Section 5 for full description of the program

CancerPEP is very similar to the Prostate Cancer PEP, a six-month home-based comprehensive health-promotion program we have developed and administered to over 250 men with prostate cancer.

CancerPEP is meant to start before a patient’s medical treatment begins, run concurrently with the treatment phase, during the recovery phase, or at time of recurrence or progression, or in long term follow-up.

The participants receive a daily email for six months that includes a description of the suggested activities for that day, as well as a link to a 3–5-minute YouTube video from the Principal Investigator (Psychology Professor) and the Study Physician (Oncologist). Participants are asked to fit the activities into their day in whatever way works best for them. The home-based activities include:

1. Aerobic Exercise: 5+ days per week – trying to meet the World Health Organization recommendations of 150-300 minutes of aerobic activity per week.
2. Strength Training or Yoga: twice per week by following 30-minute PEP YouTube videos.
3. Relaxation technique: 10 minutes per day minimum. They can watch a PEP video.
4. Dietary Advice – the daily email contains dietary and healthy eating tips.
5. Relationship training – the daily emails and PEP videos along with the weekly videos encourage the participants to connect with their loved ones
6. Optional social support – Participants have the opportunity to be ‘buddied’ up with two other co-participants with the expectation they will call each other once per week. Participants also have the option to join a biweekly zoom videoconference of other participants in the active phase of the program

## **2.5 Prostate Cancer PEP Feasibility Trial Results**

After extensive consultation with survivors, prostate cancer (PC) physicians, and health promotion experts we designed and completed a feasibility trial for a month-long version of the PEP program in February 2019 with 30 men with a history of PC.^[[1]](#footnote-2)^ Men on hormone therapy were included in this trial.

Weekly compliance to the program was measured via a weekly self-report online survey. All 30 men completed the post-intervention assessments. No participants dropped out from the program. Compliance data (the exact number of days, minutes per day and intensity of engagement in the various aspects of the program) was consistent among the four weeks with little variation noted. Overall, the order of compliance for the various aspects of the program (from the highest to the least compliance): strength and aerobic exercises, Kegels, meditation, and intimacy/connection exercises. The buddy system showed very good compliance. Twenty-five men participated in one of the three focus group sessions held after the program. No adverse events (e.g., injury) occurred.

Though the study intention was to prove the program’s feasibility and safety, there appears to have been a benefit for the participants as evidenced in the pre- and post-intervention testing: After 28-days of daily training, the group showed some physical improvements (e.g., average weight loss 3 lbs, lower diastolic blood pressure, increased flexibility) and improved current mental health assessment as measured by validated self-reported questionnaires. The K10 psychological assessment results indicated the presence of screening positive for current mental health issues in 6 men at baseline and in only 2 one month later (p=0.031). When asked whether they would recommend this same program for all new PC patients, the men gave an average recommendation score of 9.8 out of 10. Self-reported compliance measured weekly was very high throughout the 4 weeks. The feedback from the focus groups was overwhelmingly positive. Some men benefited tremendously from the program more so than what was picked up by assessing group averages.

## **2.6 Prostate Cancer PEP Phase 3 Trial Results**

The PC-PEP randomized controlled trial for men scheduled for curative treatment opened in December 2019 and completed accrual of 128 men in January 2021. Men were randomized to an ‘early’ six-month home-based PEP program or to a wait-list controlled arm in which they start the same program at the six-month point. The primary endpoint is mental health measured at six months. To date, compliance with the in-person assessments (baseline, six and 12 months) and online surveys is close to 100%. Forty participants who have completed the active portion of the program (six months of daily training) completed the exit online survey. These men rate the usefulness of the overall program ‘to them personally’ as 8.7 out of 10 and would highly recommend the program to all men scheduled for curative treatment (9.47 out of 10) ^49^. In addition, we have completed qualitative exit interviews on 25 men completing the active part of the program.

Mental distress at six months and one year as measured by the Kessler Psychological distress index was statistically significantly improved in the PC-PEP intervention group compared with the wait-list control group (who received PC-PEP starting at six months). At the six month point the control group was 3.59 times more likely to have a K10>20 (a clinically significant cut-off point indicating need for psychological treatment) compared with the intervention group ^50^.

To date no participant has died during the phase 3 trial and there have been no reported adverse medical events.

Anecdotally, the Urologists and Radiation Oncologists have received very positive feedback from their patients who were following the program. The Nova Scotia Cancer Care (Leslie Hill) program has also received extremely positive feedback by men who have been enrolled in PC-PEP and have been surveyed routinely post prostate cancer treatment in the past year by the Nova Scotia Cancer Centre.

## **2.7 Prostate Cancer PEP Phase 4 Trial**

The Prostate Cancer Patient Empowerment Program Phase 4 Implementation trial began recruitment in September 2021 and is expected to take four years to complete.. The eligibility criteria were expanded (from men scheduled for curative surgery or radiotherapy) to include all men with a diagnosis of prostate cancer with an estimated median survival of 2 years or more, and who are deemed safe to exercise. This means men with early metastatic prostate cancer are being included in the trial as long as they are deemed safe to exercise and do strength training.

As of November 2022, over 150 men have been accrued to the phase 4 trial. Each month participants are asked, via an online survey, whether they have suffered from any type of injury due to participation in the program. To date, there have been no reported adverse events, either via these online surveys or brought to the attention of the investigators otherwise.

## **2.8 Difference between PC-PEP Phase 4 and this CancerPEP Phase 2 Proposal**

PC-PEP Phase 4 is a prostate cancer specific study, while CancerPEP is open to all types of cancer. PC-PEP participants are expected to live for 2 years, while CancerPEP participants must be given an expected median survival of greater than a year, and an expectation to be well enough to complete 6 months of the program. Participants in both trials must be deemed safe to exercise and do strength training at the start of each trial - but because prostate cancer generally is a slower growing cancer than all other cancer types, we are shortening the expected median survival criteria in the CancerPEP group, with the hope of capturing QoL data for at least a year.

The second difference is only half of the CancerPEP participants will receive a Heart-Rate Variability (HRV) biofeedback during the six-month program, while the other half will receive a monitor starting at the six-month point (for six months).

### 2.8.1 Random Assignment of ‘Early’ vs ‘Late’ Access to Heart Rate Variability Monitors

As a secondary outcome, we would also like feedback as to whether the Heart Rate Variability (HRV) devices provide significant benefit and whether people will practice a relaxation technique without a monitor. If the HRV device is not a crucial component of the program then the ‘roll-out’ of the program to other countries and those with limited financial resources will be much easier – eliminating the cost of loaning a $190-230 CAD device, and the personnel time training and supporting participants.

This trial is not meant to be a non-inferiority trial. Instead, we’ll gather information about compliance, mental health, and qualitative feedback of the experience of participants

d to receive or not receive an HRV biofeedback device during the six months of the active phase of the trial.

## **2.9 Purpose of the study**

The proposed trial is an extension of our experience creating and testing a similar empowerment program for men affected by prostate cancer. The six-month prostate program has proven to be safe in the 200+ men who have completed PC-PEP.

We are now testing the feasibility and safety of two key features offered to a diverse group of cancer patients:

1. Prescribing multiple physical and social activities, and intensive daily programming for six months to potentially weaker patients, especially as participants are directing their own aerobic and strength training programs from home.
2. We are also testing the question of whether the biofeedback monitor is a necessary component of the program.

Overall, this research aims to address one of the most critical needs of cancer survivors: the development and evaluation of interventions to improve the short and long-term mental and physical health in this vulnerable population.

# **3 STUDY OBJECTIVES AND OUTCOMES**

## **3.1** **Primary Objective**

To determine the feasibility and safety of offering a six-month comprehensive home-based cancer patient empowerment program. The feasibility of the comprehensive program will be measured by:

1. The accrual rate: percentage of people who enroll in the program who are offered the program. Each of the partnering organizations and physicians who offer the program to cancer patients/survivors will provide an estimate of the number of people approached. Our research coordinator will keep a record of the number of people who contacted us with interest in the trial, and the number of participants who enrolled.
2. The attrition rate: the percentage of people who complete the program who were enrolled. Dropping out of the program is defined as contacting us to ask that they no longer want to participate and/or not completing the six-month questionnaire.
3. The compliance rate as measured by weekly self-report of the type and quantity of program activities. The participants receive an email weekly with a link to a short survey for this purpose.
4. The incidence and severity of adverse events self-reported by participants

## **3.2** **Secondary Objectives**

3.2.1 To determine whether providing stress reduction biofeedback equipment during the program changes mental health outcomes and participant experience.

Participants will be randomly assigned to receive a heart-rate variability monitor immediately to use during the six program or at the end of the program (delayed group will have access for a six-month duration starting at the six-month point). This biofeedback monitor is used at the time of the daily relaxation technique.

The mental health (measured on the K10 scale) between the immediate and delayed groups will be compared at the six-month point (i.e., before the late group has access to the monitors). All participants will also be asked about their experience of practicing a relaxation technique daily in both quantitative (survey questionnaires at the end of the 6 months assessing each aspect of the program in perceived usefulness on a scale ranging from 0 – not useful at all to me personally to 10 – extremely useful to me personally) and qualitative questions at the end of the six-month program. Twenty participants in each of the immediate and delayed groups will undergo qualitative interviews which will include an open-ended question about the HRV monitors.

- - 1. To determine the demographic, cancer type/stage, psychological, social, medical and treatment-related risk factors predicting poor mental health and quality of life at the end of the six-month program and 6 and 18 months later, all participants will be evaluated via online surveys at baseline, and at 6, 12 and 24 months (where possible) including the Kessler Psychological Distress Scale, Dyadic Assessment Scale, Screening for Distress Questionnaire, Quality of life (SF-12 and FACT-G), Perceived Illness Questionnaire, Structural-Functional Social Support Scale, The Pittsburgh Sleep Quality Index, The Sedentary Behavior Questionnaire, and

1. Self-reported cancer type, stage, and treatment
2. Baseline demographic and personality assessment
3. Exit quantitative interview including patient satisfaction
4. Exit qualitative interview

These evaluations will help determine which patients benefit most and least from the program so that the program can be modified for those at highest risk of poor mental and physical health.

# **4 SELECTION AND ENROLLMENT OF PARTICIPANTS**

## **4.1 Eligibility Criteria**

### 4.1.1 Inclusion Criteria

- Age >18.
- History of a cancer diagnosis, as per the participant’s report.
- Safe to exercise and do strength training. Participants who have recovered from a stroke or heart condition in the past 12 months will require approval from their Family Physician, Oncologist, or Cardiologist to participate in the study.
- Participants with advanced cancer (including cancer spread to bones) will need approval from the Study Physician or their Oncologist to participate.
- Existing (or willingness to create) email account and willingness to access email daily.
- Ability to follow website links to watch YouTube videos.
- Ability to understand and speak English.
- Ability to participate in low to moderate levels of physical activity and strength training.
- Ability and willingness to fill out an online survey at baseline, and 6, 12 and possibly 24 months, and a weekly compliance survey for the six months of the program.
- Deemed to have an expected survival greater than 1 year and expected to be well enough to complete the six-month training.

### 4.1.2 Exclusion Criteria

- Patients deemed unfit to participate in low to moderate level exercise e.g., including but not limited to a myocardial infarction or stroke within the last year, without approval from their Family Physician or Cardiologist that they are safe to exercise.
- Unable to access the internet and lack of a computer or smartphone to receive emails required for study intervention, or unable to click on a link to successfully watch a YouTube video.
- Those with a predicted survival less than 1 year, or not expected to be able to participate in the program for six months

## **4.2 Recruitment, Screening, and Randomization**

### 4.2.1 Recruitment Methods and Timeline

Potential participants from across Canada will be invited to join. Recruitment of potential participants will occur in multiple ways:

1. We will approach Canadian Cancer Community-based Organizations and cancer support groups who can then offer it to their members and contacts. Examples are Kidney Cancer Canada, Wellspring.ca, Leukemia and Lymphoma Society of Canada, and many more
2. Posters in Nova Scotia Cancer Centers main waiting rooms, and in clinic rooms
3. Word of mouth from participants, and through social media
4. The study physician and other physicians, offering pamphlets

Timeline: We expect to begin recruitment in late fall or early winter of 2022 and accrue 100 patients within 6 months. The filming of the daily videos will start late November 2022.

### 4.2.2 Pre-screening

1. All interested participants, whether they learn about the study through email, poster, word of mouth, or a health care professional, will be asked to visit CancerPEP.org
2. CancerPEP.org contains an overview of the study and educational videos describing the program. The website explains the steps that potential participants would need to take to participate in the trial (safety to exercise, strength training, consent process). The website provides the contact information of the Research Coordinator (RC).
3. The website will also contain a link to a pre-screening survey (hosted on REDCap). The pre-screening survey will ask questions such as the type of cancer the potential participant was diagnosed with, their province of residence, age, date of diagnosis, as well as phone number and email address for contact purposes. The survey will also contain the questions from the Physical Activity Readiness Questionnaire (PAR-Q) to assess the potential participants ability and safety to become more physically active.
4. The RC will be notified when these pre-screening surveys are submitted. The RC will review these submissions and contact all people who make a submission. Participants are not required to fill out the pre-screening and PAR-Q themselves. They have the option to contact the RC and the RC will ask the questions and complete the survey.
5. During this call, the RC will re-confirm some of the eligibility criteria and discuss with the potential participant if they are eligible or not. The RC will determine if the potential participant requires further assessment for safety to exercise. Safety to exercise will be confirmed at this stage if the potential participant answers ‘no’ to all questions on the PAR-Q AND they do not have advanced cancer. More information will be required to confirm safety to exercise and proceed with screening and consent in the following cases:
6. Participants with advanced cancer/metastatic disease: the RC will inform the potential participant that they need to receive written approval from either their family physician, oncologist, or the study physician to proceed
7. Participants who report having a stroke or myocardial infarction within the last 12 months: the RC will inform the potential participant that they need to receive written approval from either their family physician, oncologist, or the study physician to proceed
8. Participants who answer “yes” to one or more PAR-Q questions (other than stroke or MI in past 12 months): the RC will inform the potential participant that they are required to watch a short video demonstrating each of the 18 exercises that are part of the study intervention, before they can proceed with screening and consent.

All participants who fall into the descriptions of A-C above will be encouraged to seek professional guidance for exercise by contacting a qualified personal trainer or clinical exercise physiologist (CEP) before starting the program. These participants will be informed that becoming more physically active has potential risks of injury, and it is recommended that they seek guidance from a professional to further ensure safety while exercising.

1. The RC will also create a participant profile on the study’s Ripple Science database to keep a record of contact information, all contact logs, as well as track the participant’s study onboarding progress and future assessment dates.

### 4.2.3 Informed Consent

1. The RC will give a full explanation of the study that includes a description of the components of the intervention as well as what the participant is expected to do if they join the study. The RC will have an informed consent discussion that includes how the participants data will be stored and an explanation of the potential harms and benefits of participating. The RC will ask the potential participant if they have any questions. This will happen via telephone in most cases. At the participants request, this can occur via Zoom for Healthcare or in-person.
2. If the potential participant agrees to receive the information and consent form, the RC will email them a link to access the REDCap e-consent. The potential participant will be instructed to read the information and consent form thoroughly and contact the RC if the have any questions prior to signing.
3. Once the information and consent form is signed and submitted electronically, the participant will automatically receive a PDF copy of the signed consent form to their email.

### 4.2.4 Baseline Survey

Following informed consent, the participant must complete the baseline questionnaire. This survey will take the participant about 40 minutes to complete. The baseline questionnaire is emailed to the participant automatically once the e-consent form is received and the REDCap logic confirms that they have agreed to participate.

### 4.2.5 Randomization

Once the baseline survey has been completed by the participant, the RC will initiate the randomization by contacting the study PI, who maintains the randomization table. The randomization table is kept on a password protected excel file. Only the PI will have access to this table. The PI will not be involved in any of the pre-screening, consent, or baseline survey procedures. The staff conducting these procedures and the participants will be blinded until after baseline data collection is complete.

To control for the influence of mental distress and advanced cancer on the study outcomes, randomization will be stratified. First, participants will be stratified according to K10 score at baseline; 10-19 (no mental distress) or 20-50 (mild to severe mental distress). Participants will be further stratified by the presence or absence of advanced/metastatic cancer at baseline. This will create four strata, each with a permuted block size of four. The PI will access the table and assign the participant to the next available allotment within the strata they are assigned. The PI will inform the RC.

The RC lets the participant know and arranges for the participant to receive the HRV immediately or at six months, based on the allocation.

#

# **5 CLINICAL INTERVENTION (Cancer Patient Empowerment Program)**

## **5.1 Interventions and Administration**

Once participants have been randomized, the RC will arrange a virtual or in-person session to teach the participant to use the HeartMath device, for those randomized to receive the device immediately. The HRV monitors are to be returned to the RC at the end of six months of use.

Options to obtain the HRV monitor:

- - - - 1. The RC can arrange for mail or pick up.
        2. The participant can buy their own monitor.

The RC will also confirm if the participant has or requires the resistance training bands. Each participant +/- their partner needs two to three resistance bands. The RC can arrange to send them bands or they can buy them locally.

Participants will then be directed to watch the educational videos on the CancerPEP.org website including:

1. Overview video
2. How to get the best care from the medical system
3. Healthy eating / Dietary advice overview
4. Relationship training overview
5. Explanation of HRV monitor set up
6. Exercise, strength training and yoga overview

### 5.1.1 Aerobic Training Recommendations

Aerobic training instructions include the safe training principles (e.g., slowly increasing intensity and duration of workouts over time). If possible, the participants will try to work their way up to the recommended 150 minutes of moderate activity per week (including two or more strength training sessions weekly). Participants will be encouraged to exercise daily via emails and daily videos and to choose fun and social activities to maximize adherence.

Throughout the program, participants will be reminded to follow the direction provided by their health care practitioners, which overrides any of the general advice offered through the program. Participants will have access to the study CEP and study physician should they have any questions or concerns.

### 5.1.2 Strength Training and Yoga Recommendations

On Mondays or Thursdays, participants will receive an email encouraging them to do some sort of strength training that day. In the email there are links to four strength training videos varying by difficulty (exactly as in the prostate cancer program), an extremely gentle yoga video, and more advanced yoga video. The participant chooses the level of difficulty and adjusts their own program according to their ability (either easier or harder) depending on their fitness level or type/timing of cancer treatment they are undergoing.

The participants are asked to do a strength training program or yoga twice per week with a target of 30 minutes per session as part of the 150 minutes of aerobic exercise. Over time they can increase the intensity of the program on their own by increasing elastic band tension or number of sets or reps, or duration of session. The CEP will be available as a resource to the participants throughout the study duration.

*Participants Undergoing Surgery*

Any patient undergoing surgery will need to adjust their exercise program. Participants will be instructed to taper down their exercise regimen four days prior to the surgical date. Once the participants are admitted to hospital for their surgery, they will follow the directions of their Health Care Professionals about when and how to reinstitute an exercise program (typically six weeks after their operation). Gentle aerobic activities (e.g., walking) will start first, and only after they have been cleared by their surgeon can they restart the strength training sessions, for instance by slowly increasing the intensity and watching for discomfort (cue to decrease intensity).

#### **5.1.2.1 Exercise Supervision of Advanced Cancer Patients on this Study**

All patients who have proven cancer spread to the bone at the start of the trial will need to meet with the Study Physician and must be deemed safe to exercise (aerobic and strength training) by a physician prior to starting the intervention. The Study Physician will carefully review their situation, make recommendations as to how to modify the program. The strength training will be set at the lowest intensity level at the start of the program. The SP will encourage the participant to seek guidance from an exercise professional. The RC will call the participant once per month during the first 6-month intervention phase to ask if the participant had any adverse events or changes in their ability to exercise.

The participant will be instructed to inform the research team if their cancer has worsened during the intervention phase of the program, and if so, the participant will be required to get permission from a physician before continuing to exercise.

### 5.1.3 Logistics of Strength Training and Yoga program

The RC will mail a set of resistance exercise bands to each participant, and a printout of the 18 standard exercises used in the PEP program.

In this trial, we are investigating whether it’s safe for the patients to participate in strength training without providing in-person instruction. Our hypothesis is some participants are safe to follow a strength training video without needing instruction, some need to be shown the correct technique, while others will need to modify the program under the supervision of a professional/CEP. Here are the steps in identifying and directing the participants to the appropriate resources:

1. All participants will watch an overview video explaining the strength training program.
2. They will be asked to watch a short YouTube video which is a compilation of all 18 exercises in workouts A and B. They will self-screen in two ways:
   1. Whether they believe they could do all 18 exercises without discomfort
   2. Whether they are free from bone, joint, tendon or muscle problems or injury
3. Should they not pass either screening question they will need to undergo either in person and/or zoom training / modification of the strength program. They work with their own personal trainer at their own expense. The compilation YouTube video of all 18 exercises will be made available to personal trainers.
4. All participants will have ongoing access to our study physician.

###

### 5.1.4 Relaxation Technique Instruction

Participants are asked to practice a relaxation technique for 10 minutes every day during the six-month program. Half of the participants will be randomized to receive a heart rate variability (HRV) monitor produced by the HeartMath Institute to use at home throughout the 6-month intervention. The other half will receive the same device at the six month point to be loaned to them for six months. This device will allow them to have consistent biofeedback and further help them learn how to relax (increase their HRV). The monitor will be a Bluetooth compatible free unit synched to the patient’s iPhone or android device. The HeartMath Institute monitors were chosen based on their hardware and software’s reliable and frequent use in other clinical studies ^51,53,91^. Over 300 peer-reviewed studies have used HeartMath products, and the technology is based on 25 years of scientific research ^55^. The monitors were also well received by participants during the prostate cancer feasibility and randomized studies. Each patient connects a small probe to their earlobe which is painless and non-invasive. The probe detects their heart rate pattern and sends the signal to the device. The software produces an ongoing read out of the HRV and a display of a slow breathing pattern.

Whether or not they use a HRV monitor participants will receive instruction on how to practice a relaxation technique:

1. Watching a video produced by the HeartMath Institute (slow breathing, coupled with positive emotional image, and visualization of warming up the heart region)
2. Daily emails will include a link to a 5- or 10-minute meditation sequence produced by the PI and Study Physician.

### 5.1.5 Logistics of Heart Rate Variability (HRV) Monitors and Training

Each participant will be offered an HRV monitor (immediately or at the six month point according to the randomization) as a loan for the six months of the program. They will have the option of buying the monitor at the end. The RC will offer to help set up the monitor by a zoom meeting or in-person depending on the participant’s preference and offer technical support on an ongoing basis.

### 5.1.6 Healthy Lifestyle Promotion

Participants will be encouraged to follow a healthy lifestyle by:

1. Watching a video of the science underlying the PEP program which includes the modifiable risk factors for cancer like exercise, diet, smoking cessation, and healthy sleep practices. Participants who are smoking will be asked to contact their provincial smoking cessation program.
2. Daily emails and short videos include reminders to adhere to the recommended lifestyle habits including dietary tips.
3. Biweekly sessions led by the PI and Study Physician.

### 5.1.7 Smoking Cessation

In the online baseline questionnaire, participants will be asked about their smoking habits. Participants who indicated that they currently smoke or consume tobacco products will be provided with the contact information of smoking cessation program available in their province, such as Tobacco Free Nova Scotia (TFNS) or other provincial programs. TFNS offers free, confidential, personalized support to help people quit smoking and stay quit. Participants will be asked to call 8-1-1 to register with the service at the beginning of their 6-month intervention period after they watch the educational videos and receive other health promotion advice. A 2018 study found that Canadian provinces with smoking cessation initiatives for cancer patients offered a referral to 22-80% of ambulatory cancer patients who screened positive for smoking and 21-45% of those patients accepted the referral ^92^. Therefore, participants may be referred to smoking cessation services through standard medical care, in which this will act as extra support to further encourage acceptance of referral to TFNS or other provincial service.

### 5.1.8 Connection and Relationship Teaching

Participants will learn about and be encouraged to increase the multidimensional connection and intimacy in their lives by:

1. Watching a video produced by the PI and Study Physician.
2. Daily emails and short videos include reminders to express and ask for more connection in their lives. For instance, every Sunday the pilot participants were asked to call a family member or friend who lives at a distance.
3. Biweekly videoconference sessions led by the PI, Study Physician, and other participants, and others.

### 5.1.9 Social Support

To maximize compliance with an intense program and to improve the depth and breadth of social support participants have the option to:

1. Call two other co-participants each week. Co-participants are matched by demographic features. They are instructed to ask their ‘Buddies’ how they are doing with the program and to report their own compliance.
2. Join a biweekly videoconferencing session as described above.

### 5.1.10 Weekly Schedule

The content for the daily email and PEP talk will generally follow a schedule:

Every day we recommend physical activity/exercise and talk about healthy eating and/or staying more connected with loved ones.

In addition, each day of the week has an extra theme:

Monday focuses on the science of sleep, teaching participants how to improve the quality and quantity of this important pillar of health.

Tuesday: How to adopt and maintain Healthy Habits has its own proven method. Based on Stanford Professor BJ Fogg’s book *Tiny Habits*, and Dr. Wendy Wood’s *Good Habits / Bad Habits* this simple and powerful technique can help promote long-term behaviour change.

Wednesday’s curriculum draws on the science related to stress and mindfulness-based stress reduction and aims to decrease the fear of recurrence.

Thursday: We will explore different relaxation techniques and affirmations, and the role of gratitude during difficult times.

Friday will focus on relationships and how you can connect more with your loved ones.

Saturday is grocery shopping day, and time to watch the longer weekly ‘PEP Video’ (see below) +/- join the weekly videoconference.

Sunday focuses on how to develop more inner peace. Based on the secular book *Love is Letting Go of Fear*, we will follow the lessons set out by author Dr. Gerald Jampolsky including practicing forgiveness and letting go of the past.

Saturday’s Weekly PEP Video

The weekly videos provide more in-depth teaching along with sharing some of the stories of people participating in the program. Each video includes:

1. *PEP Cooking Class* - Each week we will demonstrate how to prepare one naturally delicious (easy, cheap, and nutritious!) plant-based dish.
2. *Cognitive restructuring* is a powerful way to reframe difficult thoughts and emotions. Each week we’ll use issues faced by people with a cancer diagnosis to teach this essential psychological skill.
3. *Relationship and Connection Teaching.* The Saturday PEP videos allow more in-depth presentation of subjects like types of intimacy, love languages, and ways to communicate with loved ones.

All participants will receive a link to the weekly PEP video as part of the program.

### 5.1.11 Weekly Compliance Survey

As in the prostate cancer trials, participants are asked to fill in an online survey every Sunday which captures their activities from the previous week. The survey takes 3-5 minutes to complete.

### 5.1.12 Biweekly Videoconferencing with active PEP participants

The platform is an easy-to-access platform which allows distant participants to join in on a videoconference. Participants will be encouraged to watch or listen in to the videoconference, and if they feel so inclined, to join in on the conversation. The conference will be hosted by the Study Physician and PI for one hour every second Saturday at noon AST (11am EST) and will be available to the participants in the active phase of the protocol. This conference will provide an opportunity to discuss issues arising on the trial and include tips on how to address health concerns and on how to maintain compliance with the protocol. Participants who are willing will be encouraged to share their experience of going through treatment.

*Maintaining Privacy during Videoconferences*

The Zoom videoconferencing software has a number of privacy features including:

1. Only participants who have a password can access a meeting. Each meeting has its own unique password.
2. Participants can join by telephone only and ‘listen’ to the conversation, with the option of not speaking or being seen.
3. The RC can provide a unique username to each person like a 4-digit number. The username is what appears as the participant’s name for those who decide to join by videoconference.
4. Each participant can mute their microphone and turn off or cover their camera.
5. Once the appropriate participants have joined a meeting, the meeting can be ‘locked’ so that no one else can join the meeting.
6. The Zoom software does not capture any personal information of the participant. Each person simply goes to a webpage and enters in the meeting-specific password.
7. The videoconferences will not be recorded.
8. Participants will be reminded to maintain the confidentiality of all other participants both at the start and end of each meeting.

### 5.1.13 Using Technology for Support

To increase compliance with this intense program, participants will receive a daily email outlining the activities recommended by the program for that day. These email notices will point out different resources (including video links) to help the participants with their implementation of the program’s recommendations.

### 5.1.14 Bank of Broadcasted Emails and Videos

To standardize the program for all participants, we will create a bank of 182 emails for each day of the six months of intervention. Each email is accompanied by a 3–5-minute YouTube video.

Once a participant has watched all educational videos, they will be asked to start the program on the first available Sunday. Each day, the each participant who is on the active phase of the trial will receive an email. The daily emails are distributed through REDCap. At the end of active phase (Day 1-182), the participants will no longer receive these dialy emails.

# **6 WITHDRAWAL OF PARTICIPANTS**

## **6.1 Protocol Violation**

Participants who do not comply with activities outlined in the intervention arm will remain on trial. They will be highly encouraged to complete the six- and 12-month assessments and provide their feedback on the exit interview.

## **6.2 Withdrawal of Participants**

Participants are free to withdraw from the trial at any stage without providing a reason and without consequence. If a participant withdraws from the study, any data collected on them up to that point in the study will go forward for study analysis. If a participant withdraws from the intervention but provides consent to complete subsequent follow up measurements, they will continue to attend study assessments and data will be used for intention-to-treat analysis.

# **7 ADVERSE EVENTS AND REPORTING REQUIREMENTS**

## **7.1 Adverse Events**

A clinical adverse event (AE) is any unfavourable or unintended sign or symptom associated with the study intervention and occurring in a participant assigned to the intervention. Participants will be able to self-report AEs to the study team at any time throughout the study. The chance of an adverse event on this study is expected to be low and very likely related to aerobic or strength training. All reported AEs will be recorded for type, severity, relationship to exercise program (aerobic, strength), expectedness, serious AE (yes/no), timing, action taken (if any) and outcome.

All AEs reported from the date of informed consent until 28 days after the end of the intervention are considered. AEs are also assessed by online survey once per month throughout the intervention phase (Day 1-182).

The risk of an adverse event from meditation, or social support is so low, that AEs related to these activities are not anticipated.

The study intervention does include home-based aerobic and strength exercises, which pose some possible AEs. All potential candidates will have been screened and must pass the requirement of being deemed safe to exercise. Our approach should systematically identify and screen out any individual for whom this study is contraindicated.

It is possible that the participants may experience the following expected non-serious adverse events as a result of participating in this study: musculoskeletal injury (sprain, strain), joint pain, falls, and/or muscle soreness. These will also be listed in the informed consent form and discussed with the participant prior to their signed consent. Participants will be instructed to contact us immediately if the experience symptoms/events beyond what is typically expected from exercising.

Rare but serious AEs are also possible, however unlikely. Those that may occur during the home-based program include myocardial infarction (heart attack), stroke, unconsciousness, bone fracture or other serious injury. These serious AEs, along with unanticipated adverse events not listed in the protocol or consent form but still believed to be as a result of participation in this study, will be reported according to sections 7.1.

The SP and RC will provide advice about home-based exercise including the principles of staying within personal physical limits, slowly increasing the intensity of the program over time, and reviewing the warning symptoms of heart attack and stroke.

## **7.2 Indications for Stopping Exercise**

The participants will be instructed about when to stop exercising. Absolute Indications to Stop Exercising: Suspicion of myocardial infarction, onset of angina (chest pain), signs of poor perfusion (pallor, cyanosis (blue skin), or cold and clammy skin), severe or unusual shortness of breath, central nervous system problems (incoordination, dizziness, confusion, visual problems), participants request to stop, extreme fatigue, skeletal fracture.

Relative Indications to Stop Exercising: Increasing chest pain, shortness of breath, wheezing, leg cramps (intermittent claudication), fatigue, signs or symptoms of exercise overload.

## **7.3 Reporting Requirements**

All AEs and SAEs whether reported by the participant, discovered during questioning, directly observed, or detected by other means must be recorded in the patient’s medical record and in the study’s database. Participants will be asked to ensure they have been or will be seen by a physician for full assessment and documentation of the event. All AEs will be reviewed by the Study Physician and PI. The PI is responsible for notifying the REB about the AEs in accordance with local regulations.

Reporting Deaths. All deaths on study will be reported through and including from time of consent for 13 months. The Study Physician will investigate the circumstances of the death and include a description of the adverse event in sufficient detail to allow for a complete medical assessment of the case and independent determination of the possible causality. The Study Physician and PI will promptly report these findings to the REB.

Statistical Analysis of Adverse Events: Adverse event rates will be summarized with frequency and percentage.

# **8 CLINICAL EVALUATIONS**

## **8.1 Schedule of Evaluations**

All assessments will be completed by online questionnaires

1. Baseline Demographic
2. QOL survey at baseline, 6, 12 and 24 months. Each survey takes about 20-30 minutes to complete
3. Weekly compliance survey
4. Monthly ‘safety to exercise’ survey
5. Exit quantitative survey done at 6 months
6. Qualitative Interview of first 20 participants at six months

## **8.2 Online Questionnaires**

All online questionnaires will be administered via Research Electronic Data Capture (REDCap) and they will include the following questionnaires:

1. *Demographics:* e.g., education, household income, relationship status, etc.
2. *General Health Questions:* e.g., presence of health conditions, medication usage,

etc.

1. *Cancer-Specific Questions:* e.g., date of diagnosis, cancer type and stage,

treatments to date (surgery, chemotherapy, radiation). Updated on the six-month

questionnaire.

1. *Ten-Item Personality Inventory (TIPI):* Shortened ten-item version of the Big-

Five personality dimensions which has been tested as a valid proxy for the longer

Big-Five instruments 93.

1. *Alcohol Drinking Habit –* questions from the NIH Alcohol Consumption

questionnaire 94,95.

1. *Cannabis Use and Habit*
2. *Global Adult Tobacco Survey 2020 (GATS):* This questionnaire was designed to

enhance countries’ ability to design, implement and evaluate tobacco control

interventions 96,97*.*

1. *Rapid Eating Assessment for Participants (REAP-S)*: This questionnaire tool was

designed to help healthcare members to evaluate the diet and physical activity of

individuals fast and has been shown to be valid 98,99.

1. *Modified Charlson Comorbidity Index questionnaire (CCI):* The CCI is based on a history of concomitant disease (e.g., diabetes, renal failure, cardiovascular disease, etc.) and malignancy and it was a tool initially developed and validated in women being treated for breast cancer 100. However, since its inception, it has gone on to be used in various clinical populations and has been widely administered to predict short-term outcomes 101–106.
2. *MOS Social Support Survey:* Social support through emotional, tangible, positive, and affectionate support. This questionnaire was designed through a two-year survey of patients with chronic conditions to address the multidimensionality of social support 107.
3. *Pittsburgh Sleep Quality Index (PSQI)*: This questionnaire was developed over an 18-month period to evaluate sleep quality and disturbances over a month interval through self-rated questions and has shown consistency, internal homogeneity and validity suggesting its benefit for research activities and psychiatric clinical practices 108.
4. *Sedentary Behavior Questionnaire (SBQ) for Adults*: This questionnaire was designed to assess the duration of time spent doing 9 sedentary behaviors (sitting while listening to music, sitting and talking on the phone, doing paperwork or office work, doing arts and crafts, sitting in a car, bus, or train or driving, playing a musical instrument, sitting and reading, watching television, playing computer/ video games) 109. It was shown to be valid and reliable for all items and the total scale 110.
5. *The 6-item Female Sexual Function Index (FSFI-6):* This questionnaire is a short form of the 19-item FSFI and is used to measure sexual functioning in women. It has shown reliability and validity for both fertile and infertile women which allows for quick assessment of sexual function and disfunction111,112.
6. *Sexual Health Inventory in Men (SHIM):* This questionnaire is used to assess sexual health in men and can be used to determine if they have symptoms of erectile dysfunction. The questionnaire showed continued validity and consistency among responses113,114.
7. *Quality of Life (QoL):* Questions taken from several questionnaires will be used to assess the different components of QoL:
8. Functional Assessment of Cancer Therapy-Cancer General (FACT-G) – This questionnaire has been shown to be one of the best psychometric tools for measuring the psychosocial (e.g., emotional distress, social functioning, etc.) aspect of QoL in patients with cancer^115^.
9. Short Form 12 (SF-12) Health Survey – This questionnaire will be used to assess general health QoL ^116,117^. The SF-12 has been considered the most superior generic instrument for measuring health related QoL in individuals with cancer ^116–118^.
10. Kessler Psychological Distress Scale (K10) – The K10 is a validated and

widely used clinical instrument for the assessment of psychological

symptoms, which has demonstrated high factorial and construct validity ^119,120^.

1. GAD-7 Anxiety: The GAD-7 was developed to identify potential cases of generalized anxiety disorder (GAD). It has good reliability, excellent internal consistency (Cronbach α = .92) as well as good procedural validity ^121^.
2. Dyadic Adjustment Scale (DAS-32): The DAS is designed to assess the quality of relationships in married or cohabiting couples. It is valid and has high levels of internal consistency and test-retest reliability ^122–124^.
3. Center for Epidemiologic Studies Depression Scale (CES-D): The CES-D valid and reliable measure of depressive symptomatology for cancer patients ^113,114^.
4. Satisfaction with Life Scale: the 5-item scale was designed to measure one’s perceived life satisfaction ^125^.
5. Illness Perception Questionnaire (IPQ): The IPQ was developed as a

method of assessing cognitive representations of illness and is well

validated and is reliable across different populations of chronic illnesses ^126^.

1. Questionnaire on Distress in Cancer Patients – Short Form (QSC-R10): 10-item questionnaire to assess distress in cancer patients.

Demographics characteristics (e.g., age, sexual orientation, level of education, marital status, employment status, household income, ethnic background, urban vs. rural residence), psychological (e.g., personality, illness perception, emotional well-being, cognitive functioning), social (e.g., perceived social support, relationship satisfaction, social well-being), medical and treatment-related risk factors will be used to predict differences in primary and secondary endpoints.

# **9 STATISTICAL CONSIDERATIONS**

**9.1 Sample Justification**

A sample size of 100 for two reasons. Firstly, we are accruing from a diverse group of cancer patients with different types of cancer, stages, and treatments. Having a large sample size will allow us to assess different groups of patients - in terms of feasibility and safety. Secondly, cancer is prevalent, and we are working with organizations across the country. We believe we will be able to accrue 100 patients over a few months and complete the entire trial within a couple of years.

Note, we are also randomizing the participants to receive a biofeedback monitor immediately or at the six-month point. The trial is not powered to prove there is non- inferiority between the two groups. However, the data we collect (mental distress measured quantitatively at six months, and qualitatively through interviews in both immediate and delayed groups) will provide us feedback as to whether the biofeedback equipment is an essential part of the program. If it isn't, it will be much easier to expand the program internationally and to offer the program to people with limited financial means.

## **9.2 Analysis of Primary Outcome**

The primary endpoint (feasibility) involves simple report of accrual, attrition and compliance rates, and safety by number of self-reported and otherwise discovered adverse events at 6 months.

Sample characteristics (n, frequencies, means and standard deviation) analyses will identify the percentage of patients willing to participate in the program from those solicited via the community-based organizations, demographics, cancer specific characteristics of the sample, cancer treatment modality, personality type, and comorbidities. Attrition rate will be reported (percentage of patients who completed the program vs those who enrolled). Program safety will be evaluated by providing frequency percentages with respect to the number of patients who safely completed the program (no medical incidence associated with the physical or other training aspects of the program).

Mental health sum scores (K10 questionnaire) and Questionnaire on Stress in Cancer Patients QSC-R10 will be compared between CancerPEP intervention with access to the biofeedback monitor, vs. CancerPEP intervention without access to the biofeedback monitor at the six-month following the start of the intervention using a two-level linear model analysis evaluating mean differences in the outcomes (K10 non-specific psychological distress, anxiety and depression subscales; QSC-R10 stress in cancer patients sum scores) from pre- to post-intervention. The full model will consider all the predictors and prognostic covariates (time between diagnosis and randomization, age, gender, relationship status, Charlson Commorbidity Index, treatment modality, prescribed medication for anxiety depression or both, household income, ethnicity) as fixed effects. Analyses will be conducted using IBM SPSS statistical software (Mixed Models), version 27.0. Significance levels for all analyses will be set at 2-sided, *p* < .05. A two-level linear model analysis at the end of the 6 months access to a biofeedback device monitor will also assess condition (early/immediate vs. delayed access to the biofeedback monitor) by time (pre vs. post access to the monitor).

Participants’ mental health outcomes (sum scores) at 6 months will be compared against a sample of 200+ prostate cancer patients who have completed a similar 6-month program (PC-PEP) through a two level linear model analysis evaluating mean differences in the outcomes (K10 non-specific psychological distress, anxiety and depression subscales) by group (prostate cancer PEP vs, Cancer PEP) from pre- to post-intervention, with and without covariates control.

## **9.3 Analysis of Secondary Quantitative Outcomes**

Two-level linear model analysis will examine the effect of group (CancerPEP intervention with or without a stress monitor) on secondary end points (e.g., stress, health and mental health related quality of life, smoking, drinking and cannabis habits, diet and physical activity, sedentary behaviors, social support, relationship satisfaction, life satisfaction, perception of illness, self-reported sleep quality and sleep disturbance) over time (pre-post intervention).

Multi linear modelling will assess compliance on the 5 behavioral aspects of the program over time (weekly assessments during the duration of the program).

Effectiveness of the program from the patients’ perspective will be evaluated by providing means, SD, or percentages of patients on the evaluated questions with regards to the usefulness of the program from the patients’ point of view. Qualitative interviews will also be assessed on 20 patients and evaluate the effectiveness and impediments to program participation and completion from the patients’ viewpoint will also be assessed.

|  |
| --- |

## **9.4 Analysis of Qualitative Outcomes**

Face-to-face interviews and/or focus groups will be conducted with 10 participants in each of the two randomized groups (20 participants in total). Interviews/focus groups will be conducted on Zoom and transcribed verbatim. Only audio recordings will be kept and all video recordings are deleted immediately. Thematic analysis of the data will be guided by realist evaluation processes based on a cycle of hypothesis generation, testing and refinement of Context-Mechanism- Outcome (C-M-O) configurations. We will generate C-M-O configurations using both qualitative and quantitative data (post-hoc analyses). Patient interview materials will be qualitatively analyzed by three members of the research team, independent of each other. A detailed coding procedure will be followed and codes will be compared among the raters and re-coded if necessary. Codes will be used to identify and capture themes. Data will be stored, managed, and coded with the latest NVivo qualitative software.

#

# **10 PROTECTION OF HUMAN SUBJECTS**

## **10.1 Confidentiality**

Most study data will be captured using REDCap, hosted on secure NSHA servers. This includes the data collected from all patient self-reported questionnaires. Only approved study team members with NSHA REDCap logins will have access to the data. All study team members will have signed an NSHA Pledge of Confidentiality. Data will be downloaded for analyses by the Principal Investigators, Research Coordinator, and analyzed by a Data Analyst. The computers used for data analyses are password protected and stored in locked offices within the hospital. After study closure, all study related information will be exported from REDCap and transferred and stored within the Department of Research Services for a minimum of 7 years. At the end of the retention period, the Department of Research Services will arrange for destruction in accordance with applicable standards.

## **10.2 Harms**

The majority of the practices in the protocol have no possible harms, except exercise. However, exercise has been shown to be a safe and effective means of preventing and improving a multitude of physical and psychological treatment and disease-related side- effects across the cancer trajectory (e.g., on and off treatment). For example, research has shown that cancer survivors who exercise not only have a reduced risk of disease recurrence 127 and all-cause/cancer mortality 127,128 but also have reduced side-effects of their cancer and/or its treatment such as fatigue 129–131, anxiety 132,133, depression 131,133, and cancer-related pain 134. Thus, while there is always the risk of increased short-term fatigue, stiffness, muscle soreness, and injury with any exercise program, exercise has been shown to be a safe supportive care intervention for cancer patients and survivors and the potential benefits (e.g., improved physical, social and emotional functioning and overall quality of life) outweigh the risks. Furthermore, each participant will be required to be deemed safe to exercise and to do strength training.

Participants will be asked to fill out several questionnaires regarding their health, substance use, etc. Some participants might find some of these questions uncomfortable, distressing, or upsetting to answer. Participants can choose not to answer any questions that they do not wish to answer.

Risk of a privacy/confidentiality breach data is also a potential harm. This could occur if unapproved access/disclosure, security, or cyber breach occurs, or accidental disclosure due to human, system, technological or administrative error, for example.

In addition, the participants will be told to alert the PI, SP or RC of the study via email or telephone call of any unexpected adverse effects (e.g., exercise induced, mental stress or disease progression may develop). Contact information will be provided to the participants on the first day of the study. Participants will also be encouraged at the training sessions to call and discuss their feelings being unearthed by the process if they feel they want to.

## **10.3 Benefits**

As a result of following the 6 month study intervention, participants may experience improved physical fitness, quality of life, mental health, lifestyle changes, and social connection. In addition, findings from the current study may improve our understanding of the impacts of cancer on patients’ lives and may inform best practices for programs and interventions that can improve cancer patient’s quality of life. This information will be used to improve the care at NSHA and other cancer centres in Canada.

##

## **10.4 Liability**

There are no statements in any documents that attempt to limit the liability to which the investigators or affiliated institutions are subject.

## **10.5 Disclosure of Any Financial Compensation**

Participation in the study is completely voluntarily. Participants will be given no monetary compensation for taking part in this study.

# **11 PUBLICATION OF RESEARCH FINDINGS**

## **11.1 Dissemination Plan**

We will aim to produce a study report at the end of the trial and will release the results of the study to public and media outlets. This is in addition to producing scientific manuscripts on the evaluation of CancerPEP and the contextual psychosocial determinants of QoL in community members, policy makers and support group representatives. The Soillse Scientist’s lab will post updates on a website and allow the free dissemination of study reports resulting from this initiative. Results will be presented to national and international conferences. In addition, the PI and Clinical Lead (RR) will disseminate the results through regular presentation to cancer support groups for patients and survivors as well as cancer not-for-profit organizations such as Canadian Cancer Society, Wellspring, Inspire Health, etc. The members of the research team have demonstrated effective knowledge translation and exchange practices in previous work. We will publish the results of the proposed study in high impact journals and present the results to international conferences. We will train three MSc graduates and one Undergraduate student with the proposed methods and data of this study.

#

# **LIST OF ABBREVIATIONS**

**AE –** Adverse Event

**BMI –** Body Mass Index

**CEP –** Certified Exercise Physiologist

**CancerPEP –** Cancer Patient Empowerment Program

**HRV -** Heart-Rate Variability

**K10 -** Kessler Psychological Distress Scale

**PC** – Prostate Cancer

**PC–PEP –** Prostate Cancer Patient Empowerment Program

**PI -** Principal Investigator

**QoL –** Quality of Life

**RC –** Research Coordinator

**RT -** Radiotherapy

**SP –** Study Physician

**12 REFERENCES**

1. Brenner DR, Poirier A, Woods RR, et al. Projected estimates of cancer in Canada in 2022. *CMAJ*. 2022;194(17):E601-E607. doi:10.1503/cmaj.212097
2. Mehnert A, Brähler E, Faller H, et al. Four-Week Prevalence of Mental Disorders in Patients With Cancer Across Major Tumor Entities. *JCO*. 2014;32(31):3540-3546. doi:10.1200/JCO.2014.56.0086
3. Pitman A, Suleman S, Hyde N, Hodgkiss A. Depression and anxiety in patients with cancer. *BMJ*. Published online April 25, 2018:k1415. doi:10.1136/bmj.k1415
4. Walker J, Holm Hansen C, Martin P, et al. Prevalence of depression in adults with cancer: a systematic review. *Annals of Oncology*. 2013;24(4):895-900. doi:10.1093/annonc/mds575
5. Schellekens MPJ, Lee ML. Loneliness and belonging: Exploring experiences with the COVID ‐19 pandemic in psycho‐oncology. *Psycho‐Oncology*. 2020;29(9):1399- 1401. doi:10.1002/pon.5459
6. Han J, Zhou F, Zhang L, Su Y, Mao L. Psychological symptoms of cancer survivors during the COVID‐19 outbreak: A longitudinal study. *Psycho‐Oncology*. 2021;30(3):378-384. doi:10.1002/pon.5588
7. Moraliyage H, De Silva D, Ranasinghe W, et al. Cancer in Lockdown: Impact of the COVID-19 Pandemic on Patients with Cancer. *The Oncologist*. 2021;26(2):e342- e344. doi:10.1002/onco.13604
8. Chao C, Bhatia S, Xu L, et al. Chronic Comorbidities Among Survivors of Adolescent and Young Adult Cancer. *JCO*. 2020;38(27):3161-3174. doi:10.1200/JCO.20.00722
9. Edwards BK, Noone A, Mariotto AB, et al. Annual Report to the Nation on the status of cancer, 1975‐2010, featuring prevalence of comorbidity and impact on survival among persons with lung, colorectal, breast, or prostate cancer. *Cancer*. 2014;120(9):1290-1314. doi:10.1002/cncr.28509
10. Knols R, Aaronson NK, Uebelhart D, Fransen J, Aufdemkampe G. Physical Exercise in Cancer Patients During and After Medical Treatment: A Systematic Review of Randomized and Controlled Clinical Trials. *JCO*. 2005;23(16):3830- 3842. doi:10.1200/JCO.2005.02.148
11. Galvão DA, Newton RU. Review of Exercise Intervention Studies in Cancer Patients. *JCO*. 2005;23(4):899-909. doi:10.1200/JCO.2005.06.085
12. Schmitz KH, Holtzman J, Courneya KS, Mâsse LC, Duval S, Kane R. Controlled Physical Activity Trials in Cancer Survivors: A Systematic Review and Meta-analysis. *Cancer Epidemiology, Biomarkers & Prevention*. 2005;14(7):1588-1595. doi:10.1158/1055-9965.EPI-04-0703
13. Schwartz AL. Physical Activity. *Seminars in Oncology Nursing*. 2008;24(3):164- 170. doi:10.1016/j.soncn.2008.05.004
14. Mctiernan A, Friedenreich CM, Katzmarzyk PT, et al. Physical Activity in Cancer Prevention and Survival: A Systematic Review. *Medicine & Science in Sports & Exercise*. 2019;51(6):1252-1261. doi:10.1249/MSS.0000000000001937
15. Hanson ED, Wagoner CW, Anderson T, Battaglini CL. The Independent Effects of Strength Training in Cancer Survivors: a Systematic Review. *Curr Oncol Rep*. 2016;18(5):31. doi:10.1007/s11912-016-0511-3
16. Bourke L, Smith D, Steed L, et al. Exercise for Men with Prostate Cancer: A Systematic Review and Meta-analysis. *European Urology*. 2016;69(4):693-703. doi:10.1016/j.eururo.2015.10.047
17. Courneya KS, Segal RJ, Mackey JR, et al. Effects of Aerobic and Resistance Exercise in Breast Cancer Patients Receiving Adjuvant Chemotherapy: A Multicenter Randomized Controlled Trial. *JCO*. 2007;25(28):4396-4404. doi:10.1200/JCO.2006.08.2024
18. Zhao SG, Alexander NB, Djuric Z, et al. Maintaining physical activity during head and neck cancer treatment: Results of a pilot controlled trial: Maintaining physical activity intervention in patients with head and neck cancer. *Head Neck*. 2016;38(S1):E1086-E1096. doi:10.1002/hed.24162
19. De Backer IC, Schep G, Backx FJ, Vreugdenhil G, Kuipers H. Resistance Training in Cancer Survivors: A Systematic Review. *Int J Sports Med*. 2009;30(10):703-712. doi:10.1055/s-0029-1225330
20. Strasser B, Steindorf K, Wiskemann J, Ulrich CM. Impact of Resistance Training in Cancer Survivors: A Meta-Analysis. *Medicine & Science in Sports & Exercise*. 2013;45(11):2080-2090. doi:10.1249/MSS.0b013e31829a3b63
21. Cramp F, James A, Lambert J. The effects of resistance training on quality of life in cancer: a systematic literature review and meta-analysis. *Support Care Cancer*. 2010;18(11):1367-1376. doi:10.1007/s00520-010-0904-z
22. Schumacher O, Luo H, Taaffe DR, et al. Effects of Exercise During Radiation Therapy on Physical Function and Treatment-Related Side Effects in Men With Prostate Cancer: A Systematic Review and Meta-Analysis. *International Journal of Radiation Oncology*Biology*Physics*. 2021;111(3):716-731. doi:10.1016/j.ijrobp.2021.06.034
23. Mardani Hamule M, Shahraky Vahed A. The Assessment of Relationship between Mental Health and Quality of Life in Cancer Patients. *Avicenna Journal of Clinical Medicine*. 2009;16(2):33-38.
24. Lin KY, Hu YT, Chang KJ, Lin HF, Tsauo JY. Effects of Yoga on Psychological Health, Quality of Life, and Physical Health of Patients with Cancer: A Meta- Analysis. *Evidence-Based Complementary and Alternative Medicine*. 2011;2011:1- 12. doi:10.1155/2011/659876
25. Raghavendra RM, Vadiraja HS, Nagarathna R, et al. Effects of a Yoga Program on Cortisol Rhythm and Mood States in Early Breast Cancer Patients Undergoing Adjuvant Radiotherapy: A Randomized Controlled Trial. *Integr Cancer Ther*. 2009;8(1):37-46. doi:10.1177/1534735409331456
26. Yagli NV, Ulger O. The effects of yoga on the quality of life and depression in elderly breast cancer patients. *Complementary Therapies in Clinical Practice*. 2015;21(1):7-10. doi:10.1016/j.ctcp.2015.01.002
27. Galantino ML, Desai K, Greene L, DeMichele A, Stricker CT, Mao JJ. Impact of Yoga on Functional Outcomes in Breast Cancer Survivors With Aromatase Inhibitor–Associated Arthralgias. *Integr Cancer Ther*. 2012;11(4):313-320. doi:10.1177/1534735411413270
28. Culos-Reed SN, Mackenzie MJ, Sohl SJ, Jesse MT, Zahavich ANR, Danhauer SC. Yoga & Cancer Interventions: A Review of the Clinical Significance of Patient Reported Outcomes for Cancer Survivors. *Evidence-Based Complementary and Alternative Medicine*. 2012;2012:1-17. doi:10.1155/2012/642576
29. Courneya KS. Exercise in Cancer Survivors: An Overview of Research: *Medicine & Science in Sports & Exercise*. 2003;35(11):1846-1852. doi:10.1249/01.MSS.0000093622.41587.B6
30. Hart NH, Galvão DA, Newton RU. Exercise medicine for advanced prostate cancer. *Current Opinion in Supportive & Palliative Care*. 2017;11(3):247-257. doi:10.1097/SPC.0000000000000276
31. Galvão DA, Taaffe DR, Cormie P, et al. Efficacy and safety of a modular multi- modal exercise program in prostate cancer patients with bone metastases: a randomized controlled trial. *BMC Cancer*. 2011;11(1):517. doi:10.1186/1471-2407- 11-517
32. Cormie P, Newton RU, Spry N, Joseph D, Taaffe DR, Galvão DA. Safety and efficacy of resistance exercise in prostate cancer patients with bone metastases. *Prostate Cancer Prostatic Dis*. 2013;16(4):328-335. doi:10.1038/pcan.2013.22
33. De Lazzari N, Niels T, Tewes M, Götte M. A Systematic Review of the Safety, Feasibility and Benefits of Exercise for Patients with Advanced Cancer. *Cancers*. 2021;13(17):4478. doi:10.3390/cancers13174478
34. Capozzi L, Nicole Culos-Reed. *Cancer and Exercise: Manual for Health and Fitness Professionals (4th Ed.)*. 4th ed. Thrive Health Services; 2016.
35. Carlson LE, Garland SN. Impact of mindfulness-based stress reduction (MBSR) on sleep, mood, stress and fatigue symptoms in cancer outpatients. *Int J Behav Med*. 2005;12(4):278-285. doi:10.1207/s15327558ijbm1204_9
36. Carlson LE, Ursuliak Z, Goodey E, Angen M, Speca M. The effects of a mindfulness meditation-based stress reduction program on mood and symptoms of stress in cancer outpatients: 6-month follow-up. *Support Care Cancer*. 2001;9(2):112-123. doi:10.1007/s005200000206
37. Garland SN, Carlson LE, Cook S, Lansdell L, Speca M. A non-randomized comparison of mindfulness-based stress reduction and healing arts programs for facilitating post-traumatic growth and spirituality in cancer outpatients. *Support Care Cancer*. 2007;15(8):949-961. doi:10.1007/s00520-007-0280-5
38. Lengacher CA, Johnson-Mallard V, Post-White J, et al. Randomized controlled trial of mindfulness-based stress reduction (MBSR) for survivors of breast cancer. *Psycho-Oncology*. 2009;18(12):1261-1272. doi:10.1002/pon.1529
39. Speca M, Carlson LE, Goodey E, Angen M. A Randomized, Wait-List Controlled Clinical Trial: The Effect of a Mindfulness Meditation-Based Stress Reduction Program on Mood and Symptoms of Stress in Cancer Outpatients: *Psychosomatic Medicine*. 2000;62(5):613-622. doi:10.1097/00006842-200009000-00004
40. Shapiro SL, Bootzin RR, Figueredo AJ, Lopez AM, Schwartz GE. The efficacy of mindfulness-based stress reduction in the treatment of sleep disturbance in women with breast cancer. *Journal of Psychosomatic Research*. 2003;54(1):85-91. doi:10.1016/S0022-3999(02)00546-9
41. Lamanque P, Daneault S. [Does meditation improve the quality of life for patients living with cancer?]. *Can Fam Physician*. 2006;52:474-475.
42. Kongtragul J, Tukhanon W, Tudpudsa P, et al. Effects of adding concentration therapy to Kegel exercise to improve continence after radical prostatectomy, randomized control. *J Med Assoc Thai*. 2014;97(5):513-517.
43. Mccraty R, D P, Atkinson M, Lipsenthal L. Psychological Health and Quality of Life in Patients with Diabetes.
44. Alabdulgader AA. Coherence: A Novel Nonpharmacological Modality for Lowering Blood Pressure in Hypertensive Patients. *Glob Adv Health Med*. 2012;1(2):56-64. doi:10.7453/gahmj.2012.1.2.011
45. Lehrer PM, Vaschillo E, Vaschillo B, et al. Biofeedback Treatment for Asthma. *Chest*. 2004;126(2):352-361. doi:10.1378/chest.126.2.352
46. Lehrer P, Carr RE, Smetankine A, et al. Respiratory sinus arrhythmia versus neck/trapezius EMG and incentive inspirometry biofeedback for asthma: a pilot study. *Appl Psychophysiol Biofeedback*. 1997;22(2):95-109. doi:10.1023/a:1026224211993
47. Kim S, Zemon V, Cavallo MM, Rath JF, McCraty R, Foley FW. Heart rate variability biofeedback, executive functioning and chronic brain injury. *Brain Injury*. 2013;27(2):209-222. doi:10.3109/02699052.2012.729292
48. Berry ME, Chapple IT, Ginsberg JP, Gleichauf KJ, Meyer JA, Nagpal ML. Non- pharmacological Intervention for Chronic Pain in Veterans: A Pilot Study of Heart Rate Variability Biofeedback. *Glob Adv Health Med*. 2014;3(2):28-33. doi:10.7453/gahmj.2013.075
49. Ilie G, Mason R, Bell D, et al. Development and Initial Evaluation of a Multifaceted Intervention to Improve Mental Health and Quality of Life Among Prostate Cancer Survivors. *Int J Ment Health Addiction*. 2020;18(4):1067-1080. doi:10.1007/s11469-019-00108-y
50. Ilie G, Rendon R, Mason R, MacDonald C, Kucharczyk M. A comprehensive 6- month Prostate Cancer-Patient Empowerment Program (PC-PEP) decreases psychological distress among men undergoing curative prostate cancer treatment: A Randomized Clinical Trial. *Manuscript submitted for publication*. Published online 2022.
51. Lemaire JB, Wallace JE, Lewin AM, de Grood J, Schaefer JP. The effect of a biofeedback-based stress management tool on physician stress: a randomized controlled clinical trial. *Open Med*. 2011;5(4):e154-163.
52. Karavidas MK, Lehrer PM, Vaschillo E, et al. Preliminary Results of an Open Label Study of Heart Rate Variability Biofeedback for the Treatment of Major Depression. *Appl Psychophysiol Biofeedback*. 2007;32(1):19-30. doi:10.1007/s10484-006-9029- z
53. Sarabia-Cobo CM. Heart Coherence: A New Tool in the Management of Stress on Professionals and Family Caregivers of Patients with Dementia. *Appl Psychophysiol Biofeedback*. 2015;40(2):75-83. doi:10.1007/s10484-015-9276-y
54. Bradford EJ, Wesnes KA, Brett D. Effects of peak performance training on cognitive function. *Journal of Psychopharmacology*. Published online 2005.
55. The Science of HeartMath. HeartMath. Published 2019. Accessed August 2, 2022. https://www.heartmath.com/science/
56. Victorson D, Hankin V, Burns J, et al. Feasibility, acceptability and preliminary psychological benefits of mindfulness meditation training in a sample of men diagnosed with prostate cancer on active surveillance: results from a randomized controlled pilot trial: Mindfulness and active surveillance. *Psycho-Oncology*. 2017;26(8):1155-1163. doi:10.1002/pon.4135
57. Lacroix S, Cantin J, Nigam A. Contemporary issues regarding nutrition in cardiovascular rehabilitation. *Annals of Physical and Rehabilitation Medicine*. 2017;60(1):36-42. doi:10.1016/j.rehab.2016.07.262
58. Mozaffarian D, Ludwig DS. Dietary Guidelines in the 21st Century—a Time for Food. *JAMA*. 2010;304(6):681. doi:10.1001/jama.2010.1116
59. Salminen E, Heikkilä S, Poussa T, Lagström H, Saario R, Salminen S. Female Patients Tend to Alter Their Diet Following the Diagnosis of Rheumatoid Arthritis and Breast Cancer. *Preventive Medicine*. 2002;34(5):529-535. doi:10.1006/pmed.2002.1015
60. Kassianos AP, Raats MM, Gage H, Peacock M. Quality of life and dietary changes among cancer patients: a systematic review. *Qual Life Res*. 2015;24(3):705-719. doi:10.1007/s11136-014-0802-9
61. Ravasco P, Monteiro-Grillo I, Vidal PM, Camilo ME. Dietary Counseling Improves Patient Outcomes: A Prospective, Randomized, Controlled Trial in Colorectal Cancer Patients Undergoing Radiotherapy. *JCO*. 2005;23(7):1431-1438. doi:10.1200/JCO.2005.02.054
62. Ovesen L, Allingstrup L, Hannibal J, Mortensen EL, Hansen OP. Effect of dietary counseling on food intake, body weight, response rate, survival, and quality of life in cancer patients undergoing chemotherapy: a prospective, randomized study. *JCO*. 1993;11(10):2043-2049. doi:10.1200/JCO.1993.11.10.2043
63. Loeb S, Fu BC, Bauer SR, et al. Association of plant-based diet index with prostate cancer risk. *The American Journal of Clinical Nutrition*. 2022;115(3):662-670. doi:10.1093/ajcn/nqab365
64. Ho M, Ho JWC, Fong DYT, et al. Effects of dietary and physical activity interventions on generic and cancer-specific health-related quality of life, anxiety, and depression in colorectal cancer survivors: a randomized controlled trial. *J Cancer Surviv*. 2020;14(4):424-433. doi:10.1007/s11764-020-00864-0
65. Heinrichs N, Zimmermann T, Huber B, Herschbach P, Russell DW, Baucom DH. Cancer Distress Reduction with a Couple-Based Skills Training: A Randomized Controlled Trial. *ann behav med*. 2012;43(2):239-252. doi:10.1007/s12160-011- 9314-9
66. Badger TA, Segrin C, Figueredo AJ, et al. Psychosocial interventions to improve quality of life in prostate cancer survivors and their intimate or family partners. *Qual Life Res*. 2011;20(6):833-844. doi:10.1007/s11136-010-9822-2
67. Zhang H, Zhao Q, Cao P, Ren G. Resilience and Quality of Life: Exploring the Mediator Role of Social Support in Patients with Breast Cancer. *Med Sci Monit*. 2017;23:5969-5979. doi:10.12659/MSM.907730
68. Regan TW, Lambert SD, Girgis A, Kelly B, Kayser K, Turner J. Do Couple-Based Interventions Make a Difference for Couples Affected by Cancer?: A Systematic Review. *BMC Cancer*. 2012;12(1):279. doi:10.1186/1471-2407-12-279
69. Santa Mina D, Matthew AG, Hilton WJ, et al. Prehabilitation for men undergoing radical prostatectomy: a multi-centre, pilot randomized controlled trial. *BMC Surg*. 2014;14(1):89. doi:10.1186/1471-2482-14-89
70. Aalto TJ, Malmivaara A, Kovacs F, et al. Preoperative Predictors for Postoperative Clinical Outcome in Lumbar Spinal Stenosis: Systematic Review. *Spine*. 2006;31(18):E648-E663. doi:10.1097/01.brs.0000231727.88477.da
71. Carli F, Zavorsky GS. Optimizing functional exercise capacity in the elderly surgical population: *Current Opinion in Clinical Nutrition and Metabolic Care*. 2005;8(1):23-32. doi:10.1097/00075197-200501000-00005
72. Cook JW, Pierson LM, Herbert WG, et al. The Influence of Patient Strength, Aerobic Capacity and Body Composition Upon Outcomes After Coronary Artery Bypass Grafting. *Thorac cardiovasc Surg*. 2001;49(2):89-93. doi:10.1055/s-2001- 11703
73. Singh F, Newton RU, Baker MK, et al. Feasibility of Presurgical Exercise in Men With Prostate Cancer Undergoing Prostatectomy. *Integr Cancer Ther*. 2017;16(3):290-299. doi:10.1177/1534735416666373
74. Garssen B, Boomsma MF, de Jager Meezenbroek E, et al. Stress management training for breast cancer surgery patients: SMT for breast cancer surgery patients. *Psycho-Oncology*. 2013;22(3):572-580. doi:10.1002/pon.3034
75. Cohen L, Parker PA, Vence L, et al. Presurgical Stress Management Improves Postoperative Immune Function in Men With Prostate Cancer Undergoing Radical Prostatectomy. *Psychosomatic Medicine*. 2011;73(3):218-225.
    doi:10.1097/PSY .0b013e31820a1c26
76. Liu Z, Qiu T, Pei L, et al. Two-Week Multimodal Prehabilitation Program Improves Perioperative Functional Capability in Patients Undergoing Thoracoscopic Lobectomy for Lung Cancer: A Randomized Controlled Trial. *Anesthesia & Analgesia*. 2020;131(3):840-849. doi:10.1213/ANE.0000000000004342
77. Yates P, Carter R, Cockerell R, et al. Evaluating a multicomponent survivorship programme for men with prostate cancer in Australia: a single cohort study. *BMJ Open*. 2022;12(2):e049802. doi:10.1136/bmjopen-2021-049802
78. Focht BC, Lucas AR, Grainger E, et al. Effects of a Group-Mediated Cognitive Behavioral Lifestyle Intervention on Select Social Cognitive Outcomes in Prostate Cancer Patients Undergoing Androgen Deprivation Therapy. *Integr Cancer Ther*. 2019;18:153473541989376. doi:10.1177/1534735419893764
79. Kuijpers W, Groen WG, Aaronson NK, van Harten WH. A Systematic Review of Web-Based Interventions for Patient Empowerment and Physical Activity in Chronic Diseases: Relevance for Cancer Survivors. *J Med Internet Res*. 2013;15(2):e37. doi:10.2196/jmir.2281
80. Porter LS, Fish L, Steinhauser K. Themes Addressed by Couples With Advanced Cancer During a Communication Skills Training Intervention. *Journal of Pain and Symptom Management*. 2018;56(2):252-258. doi:10.1016/j.jpainsymman.2018.04.004
81. Changizi M, Ghahremani L, Ahmadloo N, Kaveh MH. The Patient Health Engagement Model in Cancer Management: Effect of Physical Activity, Distress Management, and Social Support Intervention to Improve the Quality of Life in Breast Cancer Patients. Sahgal P, ed. *International Journal of Breast Cancer*. 2022;2022:1-8. doi:10.1155/2022/1944852
82. Hibbard JH, Greene J. What The Evidence Shows About Patient Activation: Better Health Outcomes And Care Experiences; Fewer Data On Costs. *Health Affairs*. 2013;32(2):207-214. doi:10.1377/hlthaff.2012.1061
83. Hibbard JH. Using Systematic Measurement to Target Consumer Activation Strategies. *Med Care Res Rev*. 2009;66(1_suppl):9S-27S. doi:10.1177/1077558708326969
84. Fowles JB, Terry P, Xi M, Hibbard J, Bloom CT, Harvey L. Measuring self- management of patients’ and employees’ health: Further validation of the Patient Activation Measure (PAM) based on its relation to employee characteristics. *Patient Education and Counseling*. 2009;77(1):116-122. doi:10.1016/j.pec.2009.02.018
85. Hibbard JH, Mahoney E, Sonet E. Does patient activation level affect the cancer patient journey? *Patient Education and Counseling*. 2017;100(7):1276-1279. doi:10.1016/j.pec.2017.03.019
86. Lin MY, Weng WS, Apriliyasari RW, Van Truong P, Tsai PS. Effects of Patient Activation Intervention on Chronic Diseases: A Meta-Analysis. *Journal of Nursing Research*. 2020;28(5):e116. doi:10.1097/jnr.0000000000000387
87. McCorkle R, Ercolano E, Lazenby M, et al. Self-management: Enabling and empowering patients living with cancer as a chronic illness. *CA: A Cancer Journal for Clinicians*. 2011;61(1):50-62. doi:10.3322/caac.20093
88. Small N, Bower P, Chew-Graham CA, Whalley D, Protheroe J. Patient empowerment in long-term conditions: development and preliminary testing of a new measure. *BMC Health Serv Res*. 2013;13(1):263. doi:10.1186/1472-6963-13- 263
89. Shin S, Park H. Effect of empowerment on the quality of life of the survivors of breast cancer: The moderating effect of self-help group participation: Effect of empowerment. *Jpn J Nurs Sci*. 2017;14(4):311-319. doi:10.1111/jjns.12161
90. Bulsara C, Ward A, Joske D. Haematological cancer patients: achieving a sense of empowerment by use of strategies to control illness. *J Clin Nurs*. 2004;13(2):251- 258. doi:10.1046/j.1365-2702.2003.00886.x
91. Dziembowska I, Izdebski P, Rasmus A, Brudny J, Grzelczak M, Cysewski P. Effects of Heart Rate Variability Biofeedback on EEG Alpha Asymmetry and Anxiety Symptoms in Male Athletes: A Pilot Study. *Appl Psychophysiol Biofeedback*. 2016;41(2):141-150. doi:10.1007/s10484-015-9319-4
92. Halligan M, Keen D, Timmings C, Warren GW. Results of a pan-Canadian approach to systems change for smoking cessation support in Canadian cancer centers. *JCO*. 2018;36(15_suppl):e18538-e18538. doi:10.1200/JCO.2018.36.15_suppl.e18538
93. Gosling SD, Rentfrow PJ, Swann WB. A very brief measure of the Big-Five personality domains. *Journal of Research in Personality*. 2003;37(6):504-528. doi:10.1016/S0092-6566(03)00046-1
94. Hasin D. Section 2A - ALCOHOL CONSUMPTION. :10.
95. Questionnaire | National Institute on Alcohol Abuse and Alcoholism (NIAAA). Accessed August 11, 2022. https://www.niaaa.nih.gov/research/nesarc- iii/questionnaire
96. Palipudi KM, Morton J, Hsia J, et al. Methodology of the Global Adult Tobacco Survey — 2008–2010. *Glob Health Promot*. 2016;23(2_suppl):3-23. doi:10.1177/1757975913499800
97. Global Tobacco Surveillance System. *Global Adult Tobacco Survey: Core Questionnaire with Optional Questions*. Global Adult Tobacco Survey Collaborative Group; 2020. Accessed August 4, 2022. https://cdn.who.int/media/docs/default-source/ncds/ncd- surveillance/gats/06_gats_corequestionnairewithoptionalquestions.pdf?sfvrsn=3b5c a226_15
98. Rapid Eating Assessment for Participants (REAP-S). SNAP Education Connection. Accessed August 4, 2022. https://snaped.fns.usda.gov/library/materials/rapid-eating- assessment-participants-reap-s
99. Segal-Isaacson CJ, Wylie-Rosett J, Gans KM. Validation of a short dietary assessment questionnaire: the Rapid Eating and Activity Assessment for Participants short version (REAP-S). *Diabetes Educ*. 2004;30(5):774, 776, 778 passim. doi:10.1177/014572170403000512
100. Charlson ME, Pompei P, Ales KL, MacKenzie CR. A new method of classifying prognostic comorbidity in longitudinal studies: Development and validation. Journal of Chronic Diseases. 1987;40(5):373-383. doi:10.1016/0021-9681(87)90171-8
101. Barnes DE, Mehta KM, Boscardin WJ, et al. Prediction of recovery, dependence or death in elders who become disabled during hospitalization. J Gen Intern Med. 2013;28(2):261-268. doi:10.1007/s11606-012-2226-y
102. Birim O, Maat APWM, Kappetein AP, van Meerbeeck JP, Damhuis R a. M, Bogers AJJC. Validation of the Charlson comorbidity index in patients with operated primary non-small cell lung cancer. Eur J Cardiothorac Surg. 2003;23(1):30-34. doi:10.1016/s1010-7940(02)00721-2
103. de Groot V, Beckerman H, Lankhorst GJ, Bouter LM. How to measure comorbidity. a critical review of available methods. J Clin Epidemiol. 2003;56(3):221-229. doi:10.1016/s0895-4356(02)00585-1
104. Murray SB, Bates DW, Ngo L, Ufberg JW, Shapiro NI. Charlson Index Is Associated with One-year Mortality in Emergency Department Patients with Suspected Infection. Academic Emergency Medicine. 2006;13(5):530-536. doi:10.1197/j.aem.2005.11.084
105. Singh B, Bhaya M, Stern J, et al. Validation of the Charlson comorbidity index in patients with head and neck cancer: a multi-institutional study. Laryngoscope. 1997;107(11 Pt 1):1469-1475. doi:10.1097/00005537-199711000-00009
106. Tessier A, Finch L, Daskalopoulou SS, Mayo NE. Validation of the Charlson Comorbidity Index for predicting functional outcome of stroke. Arch Phys Med Rehabil. 2008;89(7):1276-1283. doi:10.1016/j.apmr.2007.11.049
107. Sherbourne CD, Stewart AL. The MOS social support survey. Soc Sci Med. 1991;32(6):705-714. doi:10.1016/0277-9536(91)90150-b
108. Buysse DJ, Reynolds CF, Monk TH, Berman SR, Kupfer DJ. The Pittsburgh Sleep Quality Index: a new instrument for psychiatric practice and research. Psychiatry Res. 1989;28(2):193-213. doi:10.1016/0165-1781(89)90047-4
109. Sedentary Behaviour Questionnaires. The Sedentary Behaviour Research Network (SBRN). Accessed August 4, 2022. https://www.sedentarybehaviour.org/sedentary- behaviour-questionnaires/
110. Rosenberg DE, Norman GJ, Wagner N, Patrick K, Calfas KJ, Sallis JF. Reliability and validity of the Sedentary Behavior Questionnaire (SBQ) for adults. J Phys Act Health. 2010;7(6):697-705. doi:10.1123/jpah.7.6.697
111. Maroufizadeh S, Riazi H, Lotfollahi H, Omani-Samani R, Amini P. The 6-item Female Sexual Function Index (FSFI-6): factor structure, reliability, and demographic correlates among infertile women in Iran. Middle East Fertil Soc J. 2020;24(1):7. doi:10.1186/s43043-019-0008-8
112. Isidori AM, Pozza C, Esposito K, et al. ORIGINAL RESEARCH—OUTCOMES ASSESSMENT: Development and Validation of a 6-Item Version of the Female Sexual Function Index (FSFI) as a Diagnostic Tool for Female Sexual Dysfunction. The Journal of Sexual Medicine. 2010;7(3):1139-1146. doi:10.1111/j.1743- 6109.2009.01635.x
113. Bernstein AN, Levinson AW, Hobbs AR, Lavery HJ, Samadi DB. Validation of Online Administration of the Sexual Health Inventory for Men. Journal of Urology. 2013;189(4):1456-1461. doi:10.1016/j.juro.2012.10.053
114. The Sexual Health Inventory for Men (SHIM) Questionnaire. Prostate Cancer Foundation. Accessed August 9, 2022. https://www.pcf.org/c/the-sexual-health- inventory-for-men-shim-questionnaire/
115. King AJL, Evans M, Moore THM, et al. Prostate cancer and supportive care: a systematic review and qualitative synthesis of men’s experiences and unmet needs. Eur J Cancer Care (Engl). 2015;24(5):618-634. doi:10.1111/ecc.12286
116. Gandek B, Ware JE, Aaronson NK, et al. Cross-validation of item selection and scoring for the SF-12 Health Survey in nine countries: results from the IQOLA Project. International Quality of Life Assessment. J Clin Epidemiol. 1998;51(11):1171-1178. doi:10.1016/s0895-4356(98)00109-7
117. Ware JE, Sherbourne CD. The MOS 36-item short-form health survey (SF-36). I. Conceptual framework and item selection. Med Care. 1992;30(6):473-483.
118. Patrick DL, Deyo RA. Generic and disease-specific measures in assessing health status and quality of life. Med Care. 1989;27(3 Suppl):S217-232. doi:10.1097/00005650-198903001-00018
119. Kessler RC, Andrews G, Colpe LJ, et al. Short screening scales to monitor population prevalences and trends in non-specific psychological distress. Psychol Med. 2002;32(6):959-976. doi:10.1017/s0033291702006074
120. Kessler RC, Barker PR, Colpe LJ, et al. Screening for serious mental illness in the general population. Arch Gen Psychiatry. 2003;60(2):184-189. doi:10.1001/archpsyc.60.2.184
121. Spitzer RL, Kroenke K, Williams JBW, Löwe B. A Brief Measure for Assessing Generalized Anxiety Disorder: The GAD-7. Arch Intern Med. 2006;166(10):1092. doi:10.1001/archinte.166.10.1092
122. Spanier GB. Measuring Dyadic Adjustment: New Scales for Assessing the Quality of Marriage and Similar Dyads. Journal of Marriage and the Family. 1976;38(1):15. doi:10.2307/350547
123. Graham JM, Liu YJ, Jeziorski JL. The Dyadic Adjustment Scale: A Reliability Generalization Meta-Analysis. J Marriage and Family. 2006;68(3):701-717. doi:10.1111/j.1741-3737.2006.00284.x
124. Carey MP, Spector IP, Lantinga LJ, Krauss DJ. Reliability of the Dyadic Adjustment Scale. Psychological Assessment. 1993;5(2):238-240. doi:10.1037/1040-3590.5.2.238
125. Diener E, Emmons RA, Larsen RJ, Griffin S. The Satisfaction With Life Scale. Journal of Personality Assessment. 1985;49(1):71-75. doi:10.1207/s15327752jpa4901_13
126. Weinman J, Petrie KJ, Moss-morris R, Horne R. The illness perception questionnaire: A new method for assessing the cognitive representation of illness. Psychology & Health. 1996;11(3):431-445. doi:10.1080/08870449608400270
127. Lahart IM, Metsios GS, Nevill AM, Carmichael AR. Physical activity, risk of death and recurrence in breast cancer survivors: A systematic review and meta-analysis of epidemiological studies. Acta Oncol. 2015;54(5):635-654. doi:10.3109/0284186X.2014.998275
128. Scott JM, Li N, Liu Q, et al. Association of Exercise With Mortality in Adult Survivors of Childhood Cancer. JAMA Oncol. 2018;4(10):1352-1358. doi:10.1001/jamaoncol.2018.2254
129. Juvet LK, Thune I, Elvsaas IKØ, et al. The effect of exercise on fatigue and physical functioning in breast cancer patients during and after treatment and at 6 months follow-up: A meta-analysis. Breast. 2017;33:166-177. doi:10.1016/j.breast.2017.04.003
130. Mustian KM, Alfano CM, Heckler C, et al. Comparison of Pharmaceutical, Psychological, and Exercise Treatments for Cancer-Related Fatigue: A Meta- analysis. JAMA Oncol. 2017;3(7):961-968. doi:10.1001/jamaoncol.2016.6914
131. Cho MH, Dodd MJ, Cooper BA, Miaskowski C. Comparisons of Exercise Dose and Symptom Severity Between Exercisers and Nonexercisers in Women During and After Cancer Treatment. Journal of Pain and Symptom Management. 2012;43(5):842-854. doi:10.1016/j.jpainsymman.2011.05.016
132. Quist M, Adamsen L, Rørth M, Laursen JH, Christensen KB, Langer SW. The Impact of a Multidimensional Exercise Intervention on Physical and Functional Capacity, Anxiety, and Depression in Patients With Advanced-Stage Lung Cancer Undergoing Chemotherapy. Integr Cancer Ther. 2015;14(4):341-349. doi:10.1177/1534735415572887
133. Chen HM, Tsai CM, Wu YC, Lin KC, Lin CC. Randomised controlled trial on the effectiveness of home-based walking exercise on anxiety, depression and cancer- related symptoms in patients with lung cancer. Br J Cancer. 2015;112(3):438-445. doi:10.1038/bjc.2014.612
134. Griffith K, Wenzel J, Shang J, Thompson C, Stewart K, Mock V. Impact of a walking intervention on cardiorespiratory fitness, self-reported physical function, and pain in patients undergoing treatment for solid tumors. Cancer. 2009;115(20):4874-4884. doi:10.1002/cncr.24551

**Table S3.** Results of Generalized Estimating Equations Analysis for Psychological Distress (K10 ≥ 20) in the complete study cohort, n=104, with the Group by Time interaction term.

| **Variable** | **B** | **SE** | **95% CI** | **Wald χ²** | **df** | **p-value** | **OR** | **95% CI for OR** |
| --- | --- | --- | --- | --- | --- | --- | --- | --- |
| **Intercept** | -2.889 | 1.454 | [-5.739, -0.039] | 3.947 | 1 | 0.047 | 0.056 | [0.003, 0.962] |
| **Time** |  |  |  | 2.270 | 2 | 0.321 |  |  |
| Time 1 vs Time 3 | 0.714 | 0.999 | [-1.244, 2.673] | 0.511 | 1 | 0.475 | 2.043 | [0.288, 14.487] |
| Time 1 vs. Time 2 | 1.612 | 1.074 | [-0.494, 3.717] | 2.251 | 1 | 0.134 | 5.012 | [0.610, 41.159] |
| Time 2 vs Time 3 | -0.897 | 1.145 | [-3.142, 1.348] | 0.614 | 1 | 0.433 | 0.408 | [0.043, 3.848] |
| **Group** | 0.291 | 0.544 | [-0.776, 1.358] | 0.286 | 1 | 0.593 | 1.338 | [0.460, 3.888] |
| **Age** | -0.003 | 0.020 | [-0.042, 0.036] | 0.018 | 1 | 0.892 | 0.997 | [0.959, 1.037] |
| **Comorbidities** | 0.389 | 0.225 | [-0.052, 0.831] | 2.988 | 1 | 0.084 | 1.476 | [0.949, 2.295] |
| **Relationship status** | 0.165 | 0.494 | [-0.804, 1.133] | 0.111 | 1 | 0.738 | 1.179 | [0.448, 3.106] |
| **Treatment type** | -0.048 | 0.257 | [-0.551, 0.455] | 0.036 | 1 | 0.851 | 0.953 | [0.576, 1.576] |
| **Medication for anxiety, depression, or both** | 1.233 | 0.468 | [0.316, 2.151] | 6.937 | 1 | 0.008 | 3.433 | [1.371, 8.595] |
| **Months between diagnosis and trial start** | 0.004 | 0.003 | [-0.003, 0.010] | 1.166 | 1 | 0.280 | 1.004 | [0.997, 1.010] |
| **Time * Group** |  |  |  | 0.997 | 2 | 0.607 |  |  |
| Time 1 * Group (HRV monitor early) vs Time 3 | 0.239 | 0.592 | [-0.920, 1.399] | 0.163 | 1 | 0.686 | 1.270 | [0.398, 4.051] |
| Time 1 * Group (HRV monitor early) vs Time 2 | -0.414 | 0.587 | [-1.565, 0.736] | 0.498 | 1 | 0.480 | 0.661 | [0.209, 2.088] |
| Time 2 * Group (HRV monitor late) vs Time 3 | 0.653 | 0.666 | [-0.652, 1.959] | 0.962 | 1 | 0.327 | 1.922 | [0.521, 7.095] |

**Note:** Time 3 serves as the reference category for time comparisons, and the first category serves as the reference for categorical variables. Exp(B) represents the odds ratio, which provides the magnitude of effect for the predictors. Time 1 – study start; Time 2 – 6 months; Time 3 – 12 months

**Table S4:** Results of Generalized Estimating Equations Analysis for Psychological Distress (K10 ≥ 20) in Breast Cancer Cohort with the Group by Time interaction terms, n=58.

| **Variable** | **B** | **SE** | **95% CI** | **Wald χ²** | **df** | **p-value** | **OR** | **95% CI for ORs** |
| --- | --- | --- | --- | --- | --- | --- | --- | --- |
| **Intercept** | -5.118 | 2.445 | [-9.909, -0.327] | 4.383 | 1 | 0.036 | 0.006 | [0.00005, 0.721] |
| **Time** |  |  |  | 2.784 | 2 | 0.249 |  |  |
| Time 1 vs Time 3 | 1.705 | 1.318 | [-0.878, 4.287] | 1.674 | 1 | 0.196 | 5.500 | [0.416, 72.746] |
| Time 1 vs Time 2 | 1.587 | 1.208 | [-0.781,3.955] | 1.724 | 1 | 0.189 | 4.887 | [0.040,19.626] |
| Time 2 vs Time 3 | 0.118 | 1.579 | [-2.977, 3.213] | 0.006 | 1 | 0.940 | 1.125 | [0.051, 24.853] |
| **Group** | 1.314 | 0.743 | [-0.141, 2.770] | 3.131 | 1 | 0.077 | 3.722 | [0.868, 15.956] |
| **Age** | 0.026 | 0.032 | [-0.037, 0.089] | 0.668 | 1 | 0.414 | 1.027 | [0.964, 1.093] |
| **Comorbidities** | 0.422 | 0.337 | [-0.238, 1.081] | 1.570 | 1 | 0.210 | 1.525 | [0.788, 2.949] |
| **Relationship status** | 0.000 | 0.690 | [-1.353, 1.352] | 0.000 | 1 | 0.999 | 1.000 | [0.258, 3.867] |
| **Treatment type** | -0.615 | 0.567 | [-1.726, 0.495] | 1.180 | 1 | 0.277 | 0.540 | [0.178, 1.641] |
| **Medication for anxiety, depression, or both** | 1.478 | 0.671 | [0.163, 2.793] | 4.855 | 1 | 0.028 | 4.384 | [1.177, 16.326] |
| **Months between diagnosis and trial start** | 0.00002 | 0.007 | [-0.014, 0.014] | 0.000 | 1 | 0.998 | 1.000 | [0.986, 1.014] |
| **Time * Group** |  |  |  | 0.739 | 2 | 0.691 |  |  |
| Time 1 * Group (HRV monitor early) vs Time 3 | -0.448 | 0.768 | [-1.954, 1.058] | 0.340 | 1 | 0.560 | 0.639 | [0.142, 2.880] |
| Time 1 * Group (HRV monitor early) vs Time 2 | -0.495 | 0.660 | [-1.789, 0.798] | 0.564 | 1 | 0.453 | 0.609 | [0.167, 2.221] |
| Time 2 * Group (HRV monitor early) vs Time 3 | 0.047 | 0.887 | [-1.692, 1.786] | 0.003 | 1 | 0.958 | 1.048 | [0.184, 5.965] |

**Note:** Time 3 serves as the reference category for time comparisons, and the first category serves as the reference for categorical variables. Exp(B) represents the odds ratio, which provides the magnitude of effect for the predictors. Time 1 – study start; Time 2 – 6 months; Time 3 – 12 months

**Table S5:** Tests of Model Effects for Mixed Model Analysis) in the full study cohort, n=104, with the Group by Time interaction term.

| **Source** | **Wald χ²** | **df** | **p-value** |
| --- | --- | --- | --- |
| (Intercept) | 4.4 | 1 | 0.036 |
| Group | 3.7 | 1 | 0.055 |
| Time | 13 | 2 | 0.002 |
| Age | 0.003 | 1 | 0.9 |
| Comorbidities | 4.2 | 1 | 0.041 |
| Relationship status | 0.14 | 1 | 0.7 |
| **Medication for anxiety, depression, or both** | 12 | 1 | <0.001 |
| **Months between diagnosis and trial start** | 1.4 | 1 | 0.2 |
| Treatment Type | 0.087 | 1 | 0.8 |
| Group * Time | 0.88 | 2 | 0.6 |

**Table S6:** Tests of Model Effects for Mixed Model Analysis) in the breast cancer subsample, n=58, with the Group by Time interaction term.

| **Source** | **Wald χ²** | **df** | **p-value** |
| --- | --- | --- | --- |
| (Intercept) | 3.180 | 1 | 0.075 |
| Group | 7.183 | 1 | 0.007 |
| Time | 6.060 | 2 | 0.048 |
| Age | 1.701 | 1 | 0.19 |
| Comorbidities | 2.790 | 1 | 0.095 |
| Relationship status | 0.002 | 1 | 0.9 |
| **Medication for anxiety, depression, or both** | 9.168 | 1 | 0.002 |
| **Months between diagnosis and trial start** | 0.009 | 1 | 0.9 |
| Treatment Type | 1.541 | 1 | 0.2 |
| Group * Time | 0.496 | 2 | 0.8 |

1. [↑](#footnote-ref-2)
